# Bioinspired Interfacial Hydration Engineering via Metal–Organic Frameworks for Efficient Nitrate‐To‐Ammonia Conversion in Neutral Media

**DOI:** 10.1002/advs.75361

**Published:** 2026-04-17

**Authors:** Yuyin Mao, Minghui Zhang, Xiangdong Xue, Chengyu Guo, Jian Liu

**Affiliations:** ^1^ State Key Laboratory of Photoelectric Conversion and Utilization of Solar Energy Qingdao New Energy Shandong Laboratory Qingdao Institute of Bioenergy and Bioprocess Technology Chinese Academy of Sciences Qingdao P. R. China; ^2^ College of Materials Science and Engineering Qingdao University of Science and Technology Qingdao P. R. China

**Keywords:** interfacial water structure, metal–organic frameworks, microenvironment modulation, nitrate electroreduction

## Abstract

Electrocatalytic nitrate reduction to ammonia (eNO_3_RR) offers a promising pathway for sustainable ammonia synthesis but is severely impeded by sluggish proton‐coupled electron transfer kinetics and competitive hydrogen evolution, particularly at industrially relevant current densities in neutral media. Herein, we report a bioinspired interfacial hydration engineering strategy by constructing a UiO‐66‐NH_2_ metal–organic framework overlayers on copper electrodes to boost eNO_3_RR activity and selectivity. The optimized UiO‐66‐NH_2_@Cu electrode exhibits exceptional performance, achieving a Faradaic efficiency of 98.6% and an ammonia yield rate of 5.02 mmol cm^−2^ h^−1^, and sustaining an ammonia partial current density exceeding 1 A cm^−2^. Combining in situ spectroscopy and molecular dynamics simulations, we elucidate that the UiO‐66‐NH_2_ overlayer reconstructs the interfacial hydration structure and promotes the accumulation of hydrated potassium ions (K^+^·H_2_O) within the electrical double layer. Density functional theory calculations reveal that UiO‐66‐NH_2_ effectively adsorbs K^+^·H_2_O complexes, leading to preferential interfacial accumulation of this hydrated cation. Crucially, these hydrated cations function as superior proton donors compared to bulk water, significantly lowering the activation barrier for the rate‐determining ^*^NO_3_ to ^*^NHO_3_ step. This work highlights the interfacial hydration microenvironment in modulating proton‐coupled electron transfer, and provides a generalizable design paradigm for efficient electrosynthesis through microenvironment engineering.

## Graphical abstract

Inspired by Nitrite reductase, a metal–organic framework overlayer is introduced to regulate the interfacial hydration microenvironment on Cu electrodes, enriching hydrated K^+^ species that act as efficient proton donors. This strategy enables nitrate‐to‐ammonia conversion with near‐unity selectivity at industrial‐level current densities in neutral media.

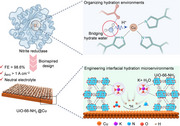

## 1 Introduction

Ammonia (NH_3_) serves as a vital chemical feedstock, extensively used in the production of fertilizers, explosives, and various industrial chemicals. More importantly, it has recently attracted growing attention as a carbon‐free hydrogen carrier for sustainable energy systems [1, 2, 3]. However, the conventional Haber–Bosch process for ammonia synthesis operates under harsh conditions (high temperature and pressure) and generates substantial CO_2_ emissions, leading to serious concerns about its high energy consumption and poor environmental sustainability. Electrochemical nitrate reduction reaction (eNO_3_RR) has emerged as a promising alternative for green ammonia synthesis. This approach not only enables decentralized green ammonia synthesis under ambient conditions but also addresses the environmental crisis of nitrate‐polluted wastewater, effectively turning a pollutant into a resource [4, 5]. Despite recent progress, achieving high selectivity and activity for NH_3_ production remains challenging due to the complex multi‐electron transfer steps, competitive side reactions (e.g., hydrogen evolution), and poorly regulated interfacial reaction microenvironments [6, 7].

Among various transition‐metal catalysts, copper has been widely recognized as an effective active material for electrochemical nitrate reduction owing to its moderate binding strength toward nitrate and key ^*^NO_x_ intermediates, which favors multi‐electron reduction pathways toward ammonia. However, the performance of Cu‐based catalysts is fundamentally constrained in neutral media, where the scarcity of free protons (H^+^ ≈ 10^−7^ m) requires water molecules to serve as the primary proton donors. As nitrate‐to‐ammonia conversion is a highly proton‐demanding process involving eight electrons and nine protons (NO_2_^−^ + 9H^+^ + 8e^−^ → NH_3_ + 3H_2_O), sluggish water activation and inefficient proton‐coupled electron transfer (PCET) severely limit reaction kinetics and selectivity [8, 9, 10, 11, 12]. Indeed, the availability of kinetic protons governs the reaction pathway and performance. Insufficient supply of protons (H^+^) or active hydrogen species (^*^H) frequently leads to the accumulation of nitrite (NO_2_^−^) intermediates, thereby compromising both the ammonia selectivity and the overall reaction rate [13, 14]. To mitigate this, prevailing strategies have focused on site engineering, such as constructing tandem dual‐metal catalysts where one component activates nitrate while the other promotes water dissociation to generate ^*^H [15, 16, 17, 18, 19, 20]. Although these strategies have achieved encouraging results, they require precise control over surface hydrogen coverage and often involve complex catalyst design. Beyond active‐site engineering, nature offers an alternative paradigm for overcoming proton‐transfer limitations without altering the catalytic center itself. In many redox enzymes, efficient proton‐coupled electron transfer is achieved through precise regulation of the secondary coordination environment surrounding the metal active site. A representative example is nitrite reductase, in which proton delivery is kinetically critical for nitrite reduction. Rather than relying on intrinsically more active metal centers, the enzyme employs positioned amino acid residues to organize local water molecules and hydrogen‐bond networks, thereby facilitating proton supply to reaction intermediates [21, 22, 23, 24, 25]. This enzymatic strategy highlights that modulation of the local hydration environment can effectively accelerate PCET and enhance catalytic efficiency, even when the primary active site remains unchanged. Recently, metal–organic frameworks (MOFs) have emerged as powerful platforms to regulate catalytic microenvironments [26, 27, 28, 29]. A successful strategy involves encapsulating active metal sites (e.g., single atoms or nanoparticles) within porous frameworks, enabling the construction of well‐defined active centers with high efficiency and selectivity for nitrate reduction to ammonia [30, 31, 32]. To further extend these concepts toward industrially relevant current densities, the construction of water‐stable MOF overlayers on highly conductive metal substrates has recently attracted increasing attention. Several studies have successfully utilized Zr‐based MOFs and its derivatives to modify Cu‐based electrodes, demonstrating remarkable eNO_3_RR performance through steric confinement and local reactant enrichment [33]. These advances have highlighted the potential of MOF‐regulated microenvironments. Nevertheless, how to rationally design MOF overlayers to regulate the interfacial hydration network, as well as how such regulation governs H generation during proton‐coupled electron transfer at the molecular level, remains largely unexplored.

**SCHEME 1:**
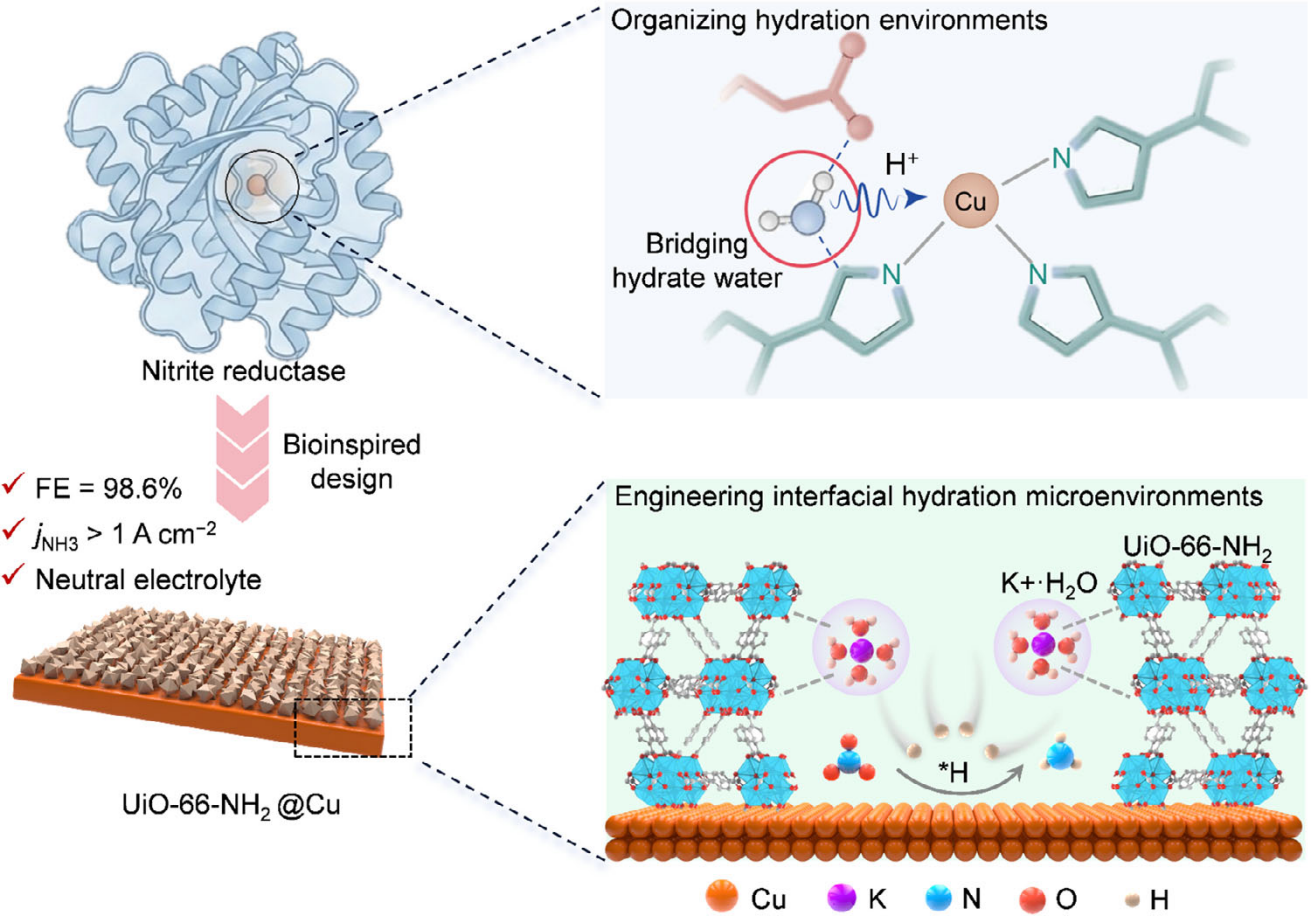
Bioinspired design of UiO‐66‐NH_2_@Cu for nitrate reduction. The UiO‐66‐NH_2_ overlayer reorganizes the interfacial hydration environment to efficiently supply protons and active hydrogen, promoting NO_3_^−^ conversion to NH_3_.

Inspired by this biological principle, we sought to regulate the interfacial microenvironment adjacent to a Cu catalytic surface to promote proton delivery for electrocatalytic nitrate reduction under neutral conditions (Scheme 1). To this end, we integrated a series of UiO‐66‐X (X = H, CH_3_, NH_2_, OH) metal–organic framework (MOF) overlayers onto Cu foil electrodes, where Cu serves as the intrinsic catalytic active site and the MOF functions as an interfacial regulator that reorganizes ion–water distributions near the electrode surface. Benefiting from its modular ligand chemistry that enables systematic functional‐group variation, the MOF overlayer offers a controllable means to regulate local ion–water interactions at the Cu interface, thereby tuning the interfacial hydration environment. Among the investigated systems, the amino‐functionalized UiO‐66‐NH_2_ framework preferentially enriches hydrated K^+^ species (K^+^·H_2_O) at the Cu interface, creating a localized hydration environment that facilitates proton donation to nitrate‐derived intermediates. This hydrated cation–mediated regulation accelerates PCET and lowers the activation barrier of nitrate hydrogenation steps, enabling highly efficient ammonia synthesis in neutral media. As a result, the UiO‐66‐NH_2_@Cu electrode delivers an exceptional ammonia Faradaic efficiency of 98.6% at industrially relevant current densities exceeding 1 A cm^−2^, together with a record‐high ammonia yield rate of 5.02 mmol cm^−2^ h^−1^. This work elucidates the pivotal role of hydrated cations in governing proton‐coupled electron transfer and establishes interfacial hydration engineering as a general design principle for achieving high‐rate and selective electrosynthesis in neutral electrolytes.

## 2 Results and Discussion

### 2.1 Catalyst Synthesis and Characterizations

**FIGURE 1:**
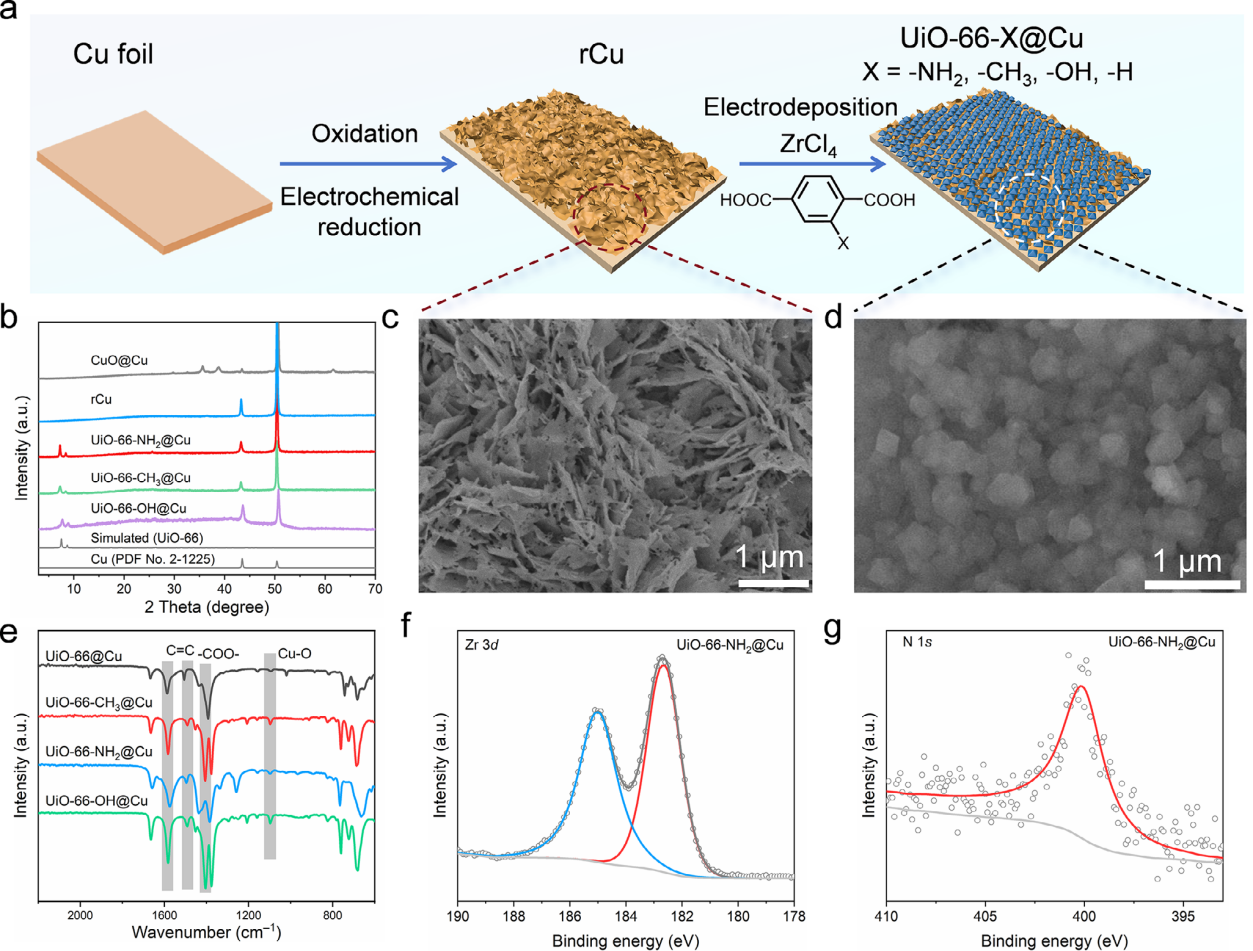
Synthesis and characterization of UiO‐66‐X@Cu. (a) Schematic illustration of the synthesis of UiO‐66‐X@Cu. (b) XRD patterns of UiO‐66‐X@Cu and roughening Cu (rCu). SEM images of (c) rCu and (d) UiO‐66‐NH_2_@Cu. (e) FT‐IR spectra of UiO‐66‐X@Cu (X = H, CH_3_, NH_2_, OH) electrodes. High‐resolution XPS spectra of the UiO‐66‐NH_2_@Cu electrode in the (f) Zr 3*d* and (g) N 1*s* regions.

The UiO‐66‐X@Cu composite electrodes were fabricated via a sequential surface reconstruction and electrochemical deposition strategy, as illustrated in Figure 1a. Initially, commercial Cu foil underwent chemical oxidation to form CuO nanosheets, followed by electrochemical reduction to generate a roughened metallic Cu substrate (noted rCu) featuring a high density of active sites. Subsequently, UiO‐66‐X overlayers were grown onto the rCu surface via a cathodic electrodeposition method, as previously reported (see Supporting Information for details) [34, 35, 36]. X‐ray diffraction (XRD, Figure 1b) analysis confirmed the formation of CuO during the oxidation process, which was subsequently reduced to metallic Cu. After UiO‐66‐X deposition, characteristic diffraction peaks of UiO‐66 in the 3°–10° region were observed, consistent with simulated patterns, indicating the successful deposition of UiO‐66‐X on the Cu substrate. Scanning electron microscopy (SEM) revealed the morphological evolution during electrode preparation. The roughened Cu foil exhibited dense nanosheet‐like Cu structures (Figure 1c), providing a high‐surface‐area scaffold for UiO‐66‐X growth. Upon electrochemical deposition, stacked UiO‐66‐NH_2_ nanocrystals formed on the surface (Figure 1d), and the corresponding optical images are shown in Figure S1. With increasing electrochemical deposition time, the growth behavior of UiO‐66‐X overlayers on the Cu substrate exhibits distinct evolution. At a short deposition time (5 min), the MOF nanoparticles were sparsely distributed, with discrete nanoparticles partially covering the Cu surface (Figure S2a). Prolonging the deposition to 20 min leads to a more continuous and uniform overlayer (Figure S2b), where the MOF crystallites coalesce into a compact layer. When the deposition time is further extended to 60 min, the overlayer becomes considerably thicker, fully encapsulating the rCu (Figure S2c). Such progressive changes in morphology directly influence catalytic behavior: thin and discontinuous overlayers offer insufficient surface modulation, while overly thick overlayers hinder mass transport and electron transfer. In contrast, the intermediate deposition time yields an optimal balance, providing intimate contact between MOF and Cu while maintaining accessible active sites and efficient charge transfer pathways. Similarly, the UiO‐66@Cu, UiO‐66‐OH@Cu, and UiO‐66‐CH_3_@Cu electrodes also exhibit densely packed UiO‐66‐X nanocrystallites (Figure S3), confirming the successful formation of continuous MOF overlayers on the Cu substrate. The characteristic vibrations of ─COO─ (1398 and 1580 cm^−1^) and C═C (1504 cm^−1^), together with the Cu─O stretching peak (1091 cm^−1^) observed in the Fourier‐transform infrared (FT‐IR, Figure 1e) spectra [37], further confirm the successful deposition of UiO‐66‐X on the Cu foil surface. Additionally, X‐ray photoelectron spectroscopy (XPS) measurements were carried out to analyze the chemical states of the electrodes. The Cu 2*p* spectra (Figure S4) of rCu reveal that a small amount of Cu^δ^^+^ remains after electrochemical reduction, likely attributed to unavoidable surface oxidation upon air exposure [38]. Crucially, distinct Zr 3*d* (Figure 1f; Figure S5) and N 1*s* (Figure 1g) signals are observed in UiO‐66@Cu and UiO‐66‐NH_2_@Cu, respectively, confirming the construction of the UiO‐66‐NH_2_ overlayer.

### 2.2 eNO_3_RR Performance Evaluation

The electrocatalytic nitrate reduction reaction (eNO_3_RR) performance of rCu and UiO‐66‐X@Cu electrodes was evaluated in a three‐electrode H‐type cell. Ammonia (Figure S6), nitrite (Figure S7), nitrate (Figure S8), and hydroxylamine(NH_2_OH, Figure S9) were quantified by UV–vis spectroscopy and ^1^H nuclear magnetic resonance spectroscopy (^1^H NMR, Figure S10). The influence of MOF deposition time on nitrate reduction performance was systematically investigated using UiO‐66@Cu as a representative system. As revealed by linear sweep voltammetry (LSV, Figure S11), the catalytic current density exhibits a volcano‐type dependence on deposition time, with an intermediate duration (20 min) delivering the highest activity. Notably, this trend is consistently reflected in both ammonia selectivity and yield rate (Figure S12). The electrode prepared with a short deposition time (5 min) exhibits a moderate NH_3_ Faradaic efficiency of 64.4% and a yield rate of 2.54 mmol h^−1^ cm^−2^, indicative of insufficient interfacial microenvironment regulation due to sparse MOF coverage. Prolonging the deposition to 20 min significantly enhances NH_3_ selectivity to 80.1% and increases the yield rate to 3.57 mmol h^−1^ cm^−2^, suggesting the establishment of an optimized interfacial hydration environment that promotes efficient proton delivery and suppresses nitrite accumulation. In contrast, further extending the deposition time to 60 min leads to a pronounced decline in both selectivity (51.2%) and yield rate (1.30 mmol h^−1^ cm^−2^), which can be attributed to reduced accessibility of Cu active sites and increased resistance to charge and reactant transport. These results demonstrate that MOF deposition time governs the degree of interfacial microenvironment establishment rather than simply altering catalyst loading, with an intermediate thickness providing the optimal balance between interfacial regulation and catalytic accessibility. Consequently, the optimized UiO‐66‐NH_2_@X (20 min) was selected for comprehensive evaluation. Furthermore, control experiments using UiO‐66‐NH_2_ drop on carbon paper (UiO‐66‐NH_2_@C) exhibited lower yield (Figure S13, 0.32 mmol h^−1^ cm^−2^) and selectivity (15.02%) compared to UiO‐66‐NH_2_@Cu and rCu, confirming that Cu acts as the primary catalytic active site.

**FIGURE 2:**
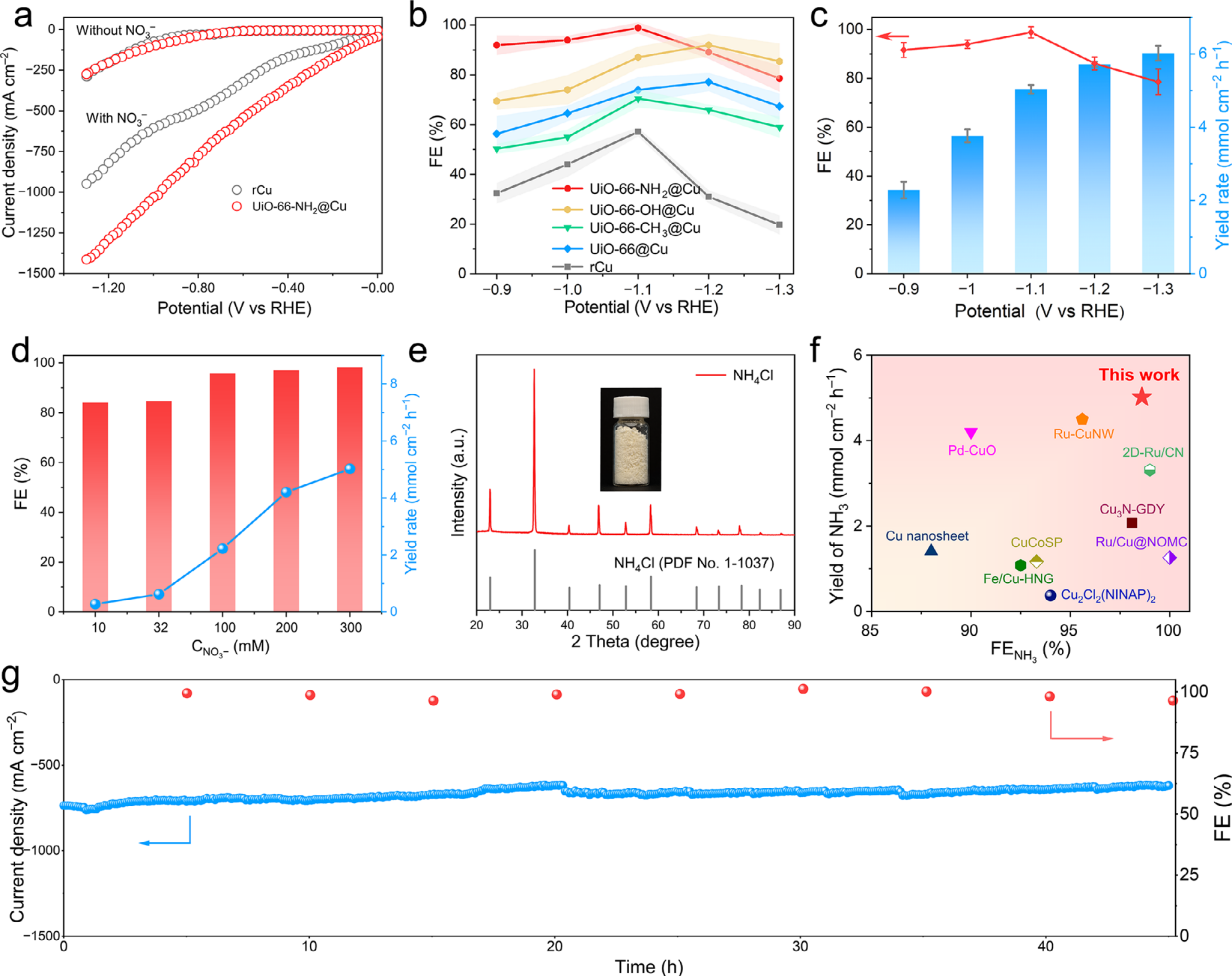
Electrocatalytic NO_3_RR performance. (a) LSV curves of UiO‐66‐NH_2_@Cu in the presence and absence of NO_3_^−^. (b) FEs of rCu, UiO‐66@Cu, UiO‐66‐CH_3_@Cu, UiO‐66‐OH@Cu, and UiO‐66‐NH_2_@Cu. (c) NH_3_ yield and FE of UiO‐66‐NH_2_@Cu under various potentials. (d) NH_3_ yield and FE of UiO‐66‐NH_2_@Cu under various NO_3_^−^ concentration. (e) XRD pattern of NH_4_Cl produced via electrocatalytic nitrate reduction. (f) Comparison of UiO‐66‐NH_2_@Cu with state‐of‐the‐art electrocatalysts in terms of NH_3_ yield and FE. (g) Continuous electrocatalytic performance of UiO‐66‐NH_2_@Cu evaluated in a continuous‐flow H‐cell using 0.3 m KNO_3_ at an applied potential of −1.1 V vs. RHE.

The catalytic behavior was assessed by LSV in neutral electrolytes. Upon the addition of nitrate, a drastic increase in current density and a positive shift in onset potential were observed (Figure 2a; Figure S14), signaling the facile initiation of nitrate reduction. Interestingly, the bare rCu electrode displays two distinct reduction features assigned to the conversion of ^*^NO_3_^−^ to NO_2_^−^ (−0.1 V vs. RHE) and the subsequent reduction of ^*^NO_2_^−^ to NH_3_ (−0.6 V vs. RHE) [39]. In contrast, the UiO‐66‐NH_2_@Cu electrode exhibits a smooth, sharply rising current profile without such bifurcation. This difference suggests that the MOF overlayer effectively accelerates the reduction of intermediates, preventing the accumulation of NO_2_^−^ and optimizing the reaction kinetics. Faradaic efficiencies (FE) and ammonia yield rates were subsequently quantified over a range of applied potentials, with the corresponding chronoamperometric responses shown in Figures S15–S17. As shown in Figure 2b, all MOF‐modified electrodes exhibit markedly enhanced NH_3_ selectivity compared to bare rCu, highlighting the general effectiveness of interfacial microenvironment regulation. However, pronounced differences emerge among MOFs with distinct functional groups. While UiO‐66@Cu, UiO‐66‐OH@Cu, and UiO‐66‐CH_3_@Cu reach maximum NH_3_ FEs of 80.1%, 91.2%, and 70.5%, respectively (Figure 2b; Figures S18 and S19), the UiO‐66‐NH_2_@Cu electrode delivers a near‐unity FE of 98.6% at −1.1 V vs. RHE. Importantly, this exceptional selectivity is maintained at industrially relevant current densities exceeding 1 A cm^−2^, corresponding to an outstanding ammonia yield rate of 5.02 mmol cm^−2^ h^−1^ (Figure 2c). These results clearly demonstrate that while MOF overlayers generally improve nitrate reduction selectivity, the amino‐functionalized UiO‐66‐NH_2_ framework provides a uniquely optimized interfacial environment for proton delivery, thereby maximizing both selectivity and reaction rate. Control experiments using blank tests (Figure S20) and isotopic labeling (Figure S21) confirm that the detected ammonia originates from nitrate reduction. This was validated by the highly consistent ammonia yields obtained from both colorimetric and ^1^H NMR quantification methods (Figure S22), as well as the characteristic doublet signal in the ^15^N isotope‐labeling NMR spectra [40, 41]. Additionally, the absence of the hydroxylamine (NH_2_OH, Figure S23) byproduct was confirmed, further demonstrating the selectivity of the electrocatalytic process. To validate the practical robustness of the catalyst, we evaluated its performance under varying nitrate concentrations. UiO‐66‐NH_2_@Cu maintains an NH_3_ selectivity above 80% across a broad concentration range of 10 to 300 mm (Figure 2d). These results demonstrate the excellent catalytic activity of the UiO‐66‐NH_2_@Cu catalyst. Furthermore, we integrated the electrocatalysis with a gas‐stripping system (Figure S24) to bridge the gap between laboratory synthesis and practical production. Following 24 h of electrolysis, high‐purity ammonium chloride (NH_4_Cl) powder was successfully recovered from the electrolyte as confirmed by XRD (Figure 2e). Impressively, compared with state‐of‐the‐art nitrate reduction catalysts (Figure 2f; Table S1), UiO‐66‐NH_2_@Cu exhibited outstanding NH_3_ selectivity and yield. Finally, the long‐term durability of the electrode was examined.

To gain deeper insights into the structural evolution of the electrode during electrolysis, we systematically examined the post‐reaction catalysts. Rather than maintaining strict structural rigidity, the UiO‐66‐NH_2_ overlayer undergoes a dynamic structural transformation under operating conditions. Specifically, XRD patterns (Figure S25) revealed a substantial decrease in the MOF diffraction peaks within just 1 h of the reaction, and the characterization data collected after the 45 h stability test confirmed its amorphization. Consistent with the XRD results, SEM images (Figure S26) showed that the initially regular MOF particles became noticeably agglomerated and fused. Despite this pronounced structural evolution, the catalyst maintains a high Faradaic efficiency (>95%) for NH_3_ production over 45 h of continuous operation (Figure 2g), indicating that the catalytic functionality is preserved under working conditions. To probe the origin of this structural evolution, inductively coupled plasma (ICP, Table S2) analysis was performed on the post‐reaction electrolyte. Only trace amounts of Cu and Zr were detected in solution, indicating minimal dissolution of the metal nodes. Combined with previous literature reports [31], this observation suggests that the structural transformation is primarily associated with the dissociation and leaching of organic ligands rather than the degradation of the metal nodes. This interpretation is further supported by FT‐IR spectroscopy (Figure S27), where the intensity of ligand‐related vibrations (1400–1600 cm^−1^) decreases significantly after 1 h of electrolysis, whereas the intensity of the Zr─O bonds (600–700 cm^−1^) exhibits no obvious attenuation. Importantly, the ligand loss is not complete. Even after 45 h of continuous operation, characteristic ligand‐related absorption bands remain detectable in the FT‐IR spectra. This behavior is likely associated with the integrated overlayer architecture, where the MOF film is directly anchored on the Cu substrate, thereby suppressing the complete diffusion of ligands into the electrolyte. As a result, the catalyst evolves into a reconstructed amorphous interfacial layer containing residual ligand‐derived components. This partial retention of the organic ligands dictates the differences in catalytic activities observed among MOFs constructed with varying ligands.

### 2.3 Investigation Into the Mechanism of eNO_3_RR

To elucidate the origin of the markedly enhanced nitrate‐to‐ammonia conversion on UiO‐66‐NH_2_@Cu, we systematically investigated the proton‐coupled electron transfer (PCET) kinetics, active hydrogen availability, and interfacial hydration structure. First, the double‐layer capacitance (C_dl_), determined from cyclic voltammetry (CV) in the non‐Faradaic region (Figure S28), was used to estimate the electrochemically active surface area (ECSA). The results (Figure S29) reveal that rCu, UiO‐66@Cu, and UiO‐66‐NH_2_@Cu exhibit comparable C_dl_ values. This finding indicates that the performance differences can be directly attributed to variations in the interfacial microenvironment rather than changes in the number of accessible Cu active sites. Electrochemical kinetic analyses reveal that UiO‐66‐NH_2_@Cu exhibits substantially accelerated reaction kinetics. The Tafel slope derived from linear sweep voltammetry (LSV) fitting (Figure S30) provides a direct measure of the catalytic kinetics. UiO‐66‐NH_2_@Cu exhibits a markedly smaller Tafel slope (127 mV dec^−1^) compared to the rCu (172 mV dec^−1^) and UiO‐66@Cu (215 mV dec^−1^). This indicates a more favorable kinetic pathway that accelerates the multi‐electron reduction of nitrate. Furthermore, electrochemical impedance spectroscopy (EIS) shows a significantly reduced charge‐transfer resistance for UiO‐66‐NH_2_@Cu at the operating potential (Figure S31), reflecting more efficient interfacial charge and proton transport that promotes the conversion of nitrate‐derived intermediates.

**FIGURE 3:**
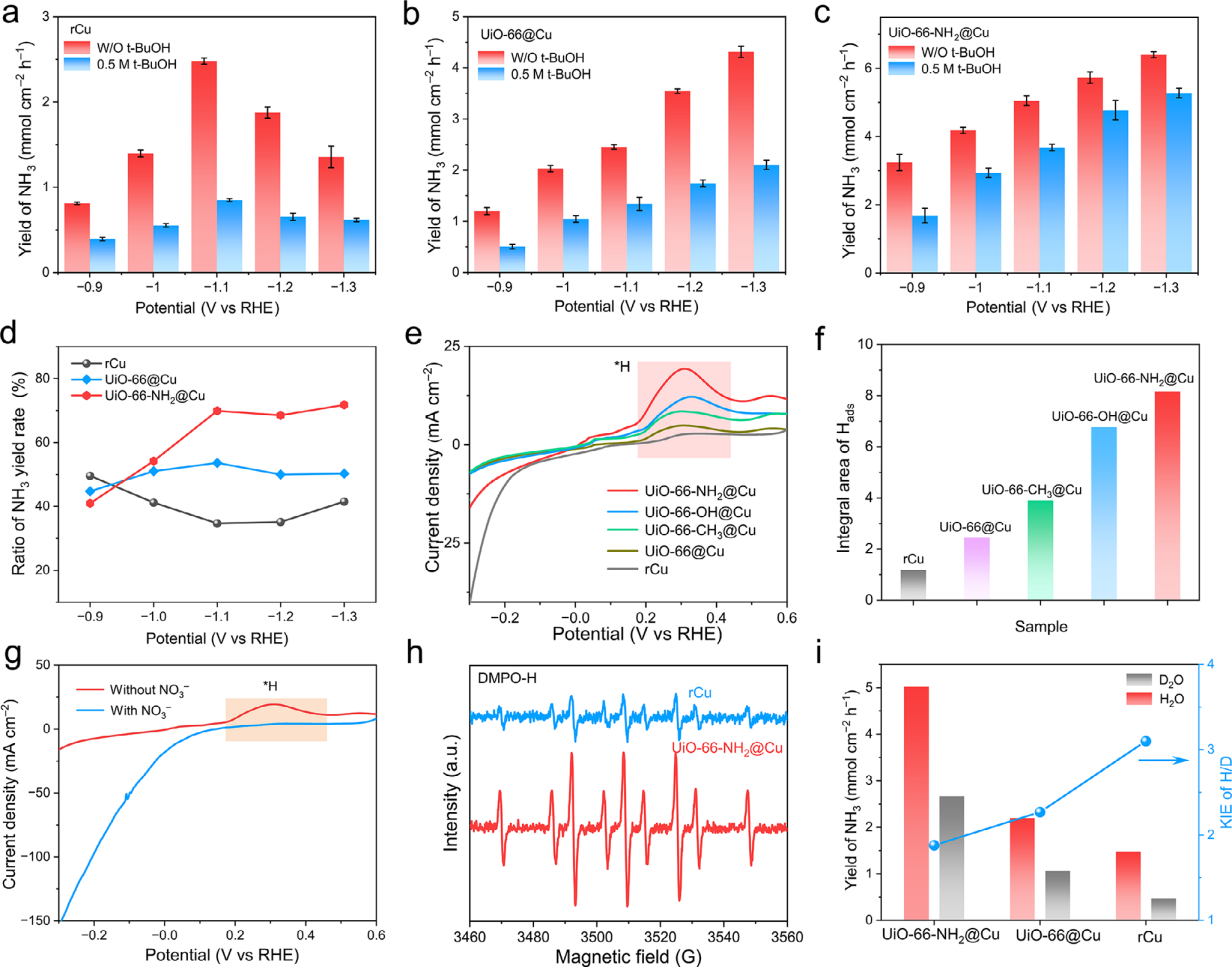
Investigation on active‐hydrogen‐promoted nitrate reduction. NH_3_ yield rates of (a) rCu, (b) UiO‐66@Cu, and (c) UiO‐66‐NH_2_@Cu under potential range from −0.9 to −1.3 V vs. RHE with and without 0.5 m t‐BuOH quencher. (d) Ratio of ammonia yield for different samples with and without t‐BuOH. (e) Hydrogen adsorption peaks of UiO‐66‐X@Cu and Cu samples in cyclic voltammetry (CV) measurements conducted in 0.5 m K_2_SO_4_ solution. (f) Integrated hydrogen adsorption peak areas of different samples derived from the CV curves. (g) CV curves of UiO‐66‐NH_2_@Cu measured in the presence and absence of 0.3 m NO_3_^−^. (h) ESR spectra of DMPO‐H over rCu and UiO‐66‐NH_2_@Cu in the absence of NO_3_^−^. (i) KIE of H/D over UiO‐66‐NH_2_@Cu, UiO‐66@Cu, and rCu.

Given that nitrate reduction in neutral media is a proton‐coupled electron transfer process, the generation of active hydrogen (^*^H) plays a pivotal role in determining the reaction rate [42, 43, 44]. Accordingly, radical quenching experiments with tert‐butanol (t‐BuOH) were performed to examine how UiO‐66‐X overlayers regulate the generation and participation of reactive hydrogen species during nitrate reduction (Figure 3a–c). Upon the addition of tert‐butanol, the catalytic activities of all samples decreased significantly, highlighting the critical role of ^*^H in nitrate reduction. Notably, UiO‐66‐NH_2_@Cu exhibits the smallest relative activity loss upon *H quenching (Figure 3d), indicating a more robust proton supply at the interface. In addition, cyclic voltammetry (CV) measurements were conducted to probe the surface concentration of adsorbed *H species. As shown in Figure 3e,f, a pronounced ^*^H adsorption feature appears in the potential window of 0.2–0.4 V vs RHE [12], with the peak area following the order: UiO‐66‐NH_2_@Cu > UiO‐66‐OH@Cu > UiO‐66‐CH_3_@Cu > UiO‐66@Cu > rCu. Despite similar ECSA values, this functional‐group‐dependent trend highlights that amino‐functionalization most effectively promotes the formation of surface‐reactive hydrogen species. Upon the addition of NO_3_^−^, the hydrogen adsorption peak decreases markedly(Figure 3g), directly evidencing the participation of these hydrogen species in nitrate hydrogenation. Furthermore, the presence of ^*^H species was directly confirmed by electron spin resonance (ESR) spectroscopy using DMPO as a spin‐trapping agent [45]. As shown in Figure 3h, the ESR spectrum displays the characteristic nine‐line hyperfine splitting signal of the DMPO‐H adduct, verifying the generation of ^*^H [46, 47, 48]. More importantly, the markedly stronger signal intensity observed for UiO‐66‐NH_2_@Cu compared with rCu indicates its superior capability for water dissociation and generation of active hydrogen species. To directly assess the involvement of proton transfer in the rate‐determining step, kinetic isotope effect (KIE) measurements were conducted by replacing H_2_O with D_2_O. All electrodes display reduced ammonia yields in D_2_O, underscoring the pivotal role of proton transfer in nitrate reduction. Notably, UiO‐66‐NH_2_@Cu exhibits a substantially smaller KIE (1.88) than rCu (3.10) and UiO‐66@Cu (2.27), indicating that proton delivery is less kinetically limiting on the MOF‐modified interface (Figure 3i). Collectively, these results demonstrate that UiO‐66‐NH_2_@Cu sustains enhanced proton availability under neutral conditions, thereby accelerating PCET without altering the intrinsic Cu active sites.

### 2.4 In Situ Spectroscopic Analysis of Interfacial Water Structure

**FIGURE 4:**
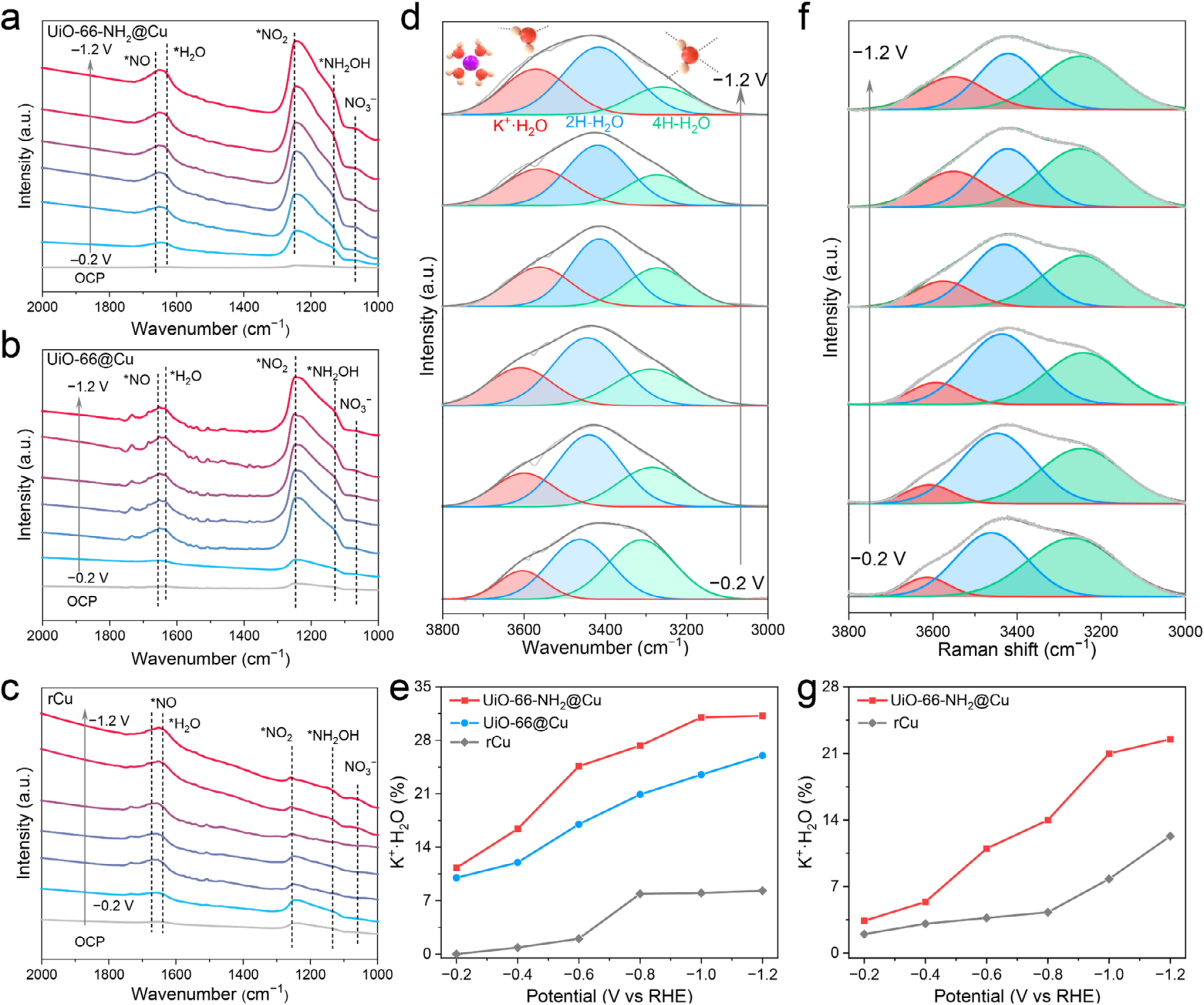
In situ spectroscopic characterization of reaction intermediates and interfacial water. In situ FT‐IR spectra of (a) UiO‐66‐NH_2_@Cu, (b) UiO‐66@Cu, and (c) rCu at different applied potentials. (d) Gaussian‐fitted peaks of in situ FT‐IR spectra revealing the three O─H stretching modes of interfacial water on UiO‐66‐NH_2_@Cu in the eNO_3_RR process. (e) Proportions of K^+^·H_2_O on the surfaces of UiO‐66@Cu, UiO‐66‐NH_2_@Cu, and Cu electrodes calculated from the peak areas of in situ FT‐IR spectra. (f) In situ Raman spectra of UiO‐66‐NH_2_@Cu under different potentials. (g) Proportions of K^+^·H_2_O on the surfaces of UiO‐66@Cu and rCu electrodes calculated from the peak areas of in situ Raman spectra.

To gain molecular‐level insights into how the UiO‐66‐NH_2_ overlayer modulates proton availability, in situ Fourier‐transform infrared (in situ FT‐IR) spectroscopy was employed. As shown in Figure 4a, the vibrational band at 1246 cm^−1^ is attributed to the adsorbed ^*^NO_2_ intermediate [49, 50], whose intensity increases with decreasing potential, indicating its pivotal role in the nitrate reduction reaction. In addition, characteristic bands corresponding to free NO_3_^−^ (1050 cm^−1^) [51], ^*^NO (1658 cm^−1^) [52, 53, 54], and ^*^NH_2_OH (1130 cm^−1^) [55, 56] intermediates were also detected, which are formed via sequential hydrogenation and deoxygenation of ^*^NO_2_. These observations suggest that the reaction pathway for nitrate reduction to ammonia proceeds through the sequence: ^*^NO_3_^−^ → ^*^NO_2_ → ^*^NO → ^*^NH_2_OH → ^*^NH_3_. Similarly, UiO‐66@Cu (Figure 4b) and rCu (Figure 4c) electrodes exhibit the same absorption features but with significantly reduced intensity. This implies that the modification with UiO‐66‐X overlayers does not alter the intrinsic reaction pathway. The observed differences in catalytic activity are more likely attributed to the modulation of interfacial microenvironments surrounding the Cu active sites rather than changes in the reaction mechanism.

In a neutral electrolyte, the hydrogen species involved in the hydrogenation of NO_3_^−^ are mainly derived from interfacial water adsorbed on the catalyst surface. Depending on the binding strength, interfacial water can be categorized into tetra‐coordinated hydrogen‐bonded water (4HB‐H_2_O), di‐coordinated hydrogen‐bonded water (2HB‐H_2_O), and alkali cation–coordinated water (K^+^·H_2_O). Among these species, K^+^·H_2_O is generally considered more weakly hydrogen‐bonded and therefore more readily activated to participate in proton transfer and generate reactive hydrogen species [57, 58]. To probe how the UiO‐66‐NH_2_ overlayer influences the distribution of interfacial water, in situ FT‐IR spectroscopy was conducted in the O─H stretching region (3000–3800 cm^−1^). As shown in Figure 4d, the in situ FT‐IR spectra of UiO‐66‐NH_2_@Cu can be deconvoluted into three components corresponding to 4HB‐H_2_O (around 3230 cm^−1^), 2HB‐H_2_O (around 3440 cm^−1^), and K^+^·H_2_O (around 3660 cm^−1^) [59, 60]. Notably, the proportion of K^+^·H_2_O on the UiO‐66‐NH_2_@Cu surface increases markedly with increasingly negative potentials, rising from 11.3% to 31.2%, indicating progressive enrichment of proton‐reactive hydration species under operating conditions. Although similar trends are observed for rCu and UiO‐66@Cu (Figures S32 and S33), the absolute proportion of K^+^·H_2_O on UiO‐66‐NH_2_@Cu (31.2%) is significantly higher than that on rCu (26.0%) and UiO‐66@Cu (8.3%, Figure 4e). Complementary in situ Raman spectroscopy corroborates this conclusion [61, 62, 63], as shown in Figure 4f and Figure S34, where the UiO‐66‐NH_2_@Cu electrode (22.5%) exhibits a significantly higher fraction of K^+^·H_2_O compared to the rCu electrode (12.2%, Figure 4g). To further verify this conclusion, K^+^ retention experiments were conducted to investigate K^+^ adsorption within the electrical double layer. As shown in Figure S35, the amount of adsorbed K^+^ follows the order UiO‐66‐NH_2_@Cu > UiO‐66@Cu > rCu, directly demonstrating that the MOF overlayer, particularly with amino functionalization, facilitates interfacial K^+^ enrichment. Collectively, these spectroscopic and electrochemical results establish that the UiO‐66‐NH_2_ overlayer does not alter the fundamental NO_3_^−^ reduction pathway on Cu, but instead reconstructs the interfacial hydration environment. By enriching K^+^·H_2_O species at the electrode surface, UiO‐66‐NH_2_@Cu promotes the formation of proton‐reactive hydration structures, thereby enhancing local proton availability and accelerating nitrate hydrogenation toward ammonia under neutral conditions.

### 2.5 Molecular Dynamics and DFT Simulations

**FIGURE 5:**
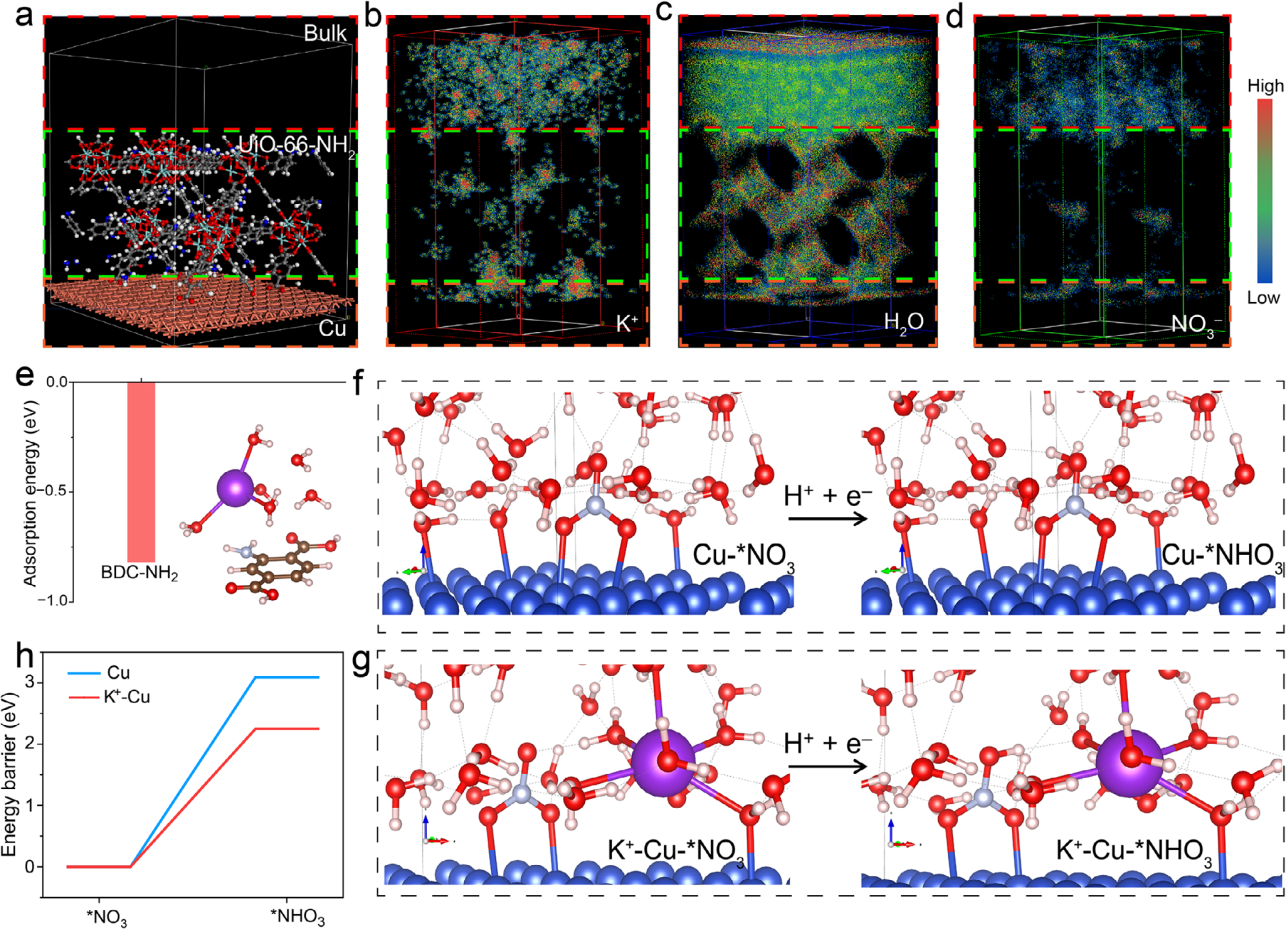
Molecular dynamics simulations and DFT calculations. (a) The model of molecular dynamics simulations. The distribution of (b) K^+^, (c) H_2_O, and (d) NO_3_^−^ on the surface of UiO‐66‐NH_2_@Cu. (e) Adsorption energy of K^+^·H_2_O on the BDC–NH_2_ ligand; the inset shows the optimized adsorption configuration. Optimized structural models illustrating the adsorption configurations of ^*^NO_3_ and ^*^NHO_3_ on the Cu surface in the (f) absence and (g) presence of K^+^·H_2_O. The red, brown, purple, baby blue, and light pink spheres represent O, C, K, N, and H atoms, respectively. (h) Energy barriers of the rate‐determining step (*NO_3_ → *NHO_3_) for NO_3_^−^ reduction on the electrode surface with and without K^+^ adsorption.

To further elucidate the role of the UiO‐66‐NH_2_ overlayer on the electrode surface, molecular dynamics (MD) simulations were performed to probe the spatial distribution of molecules and ions at the electrode–electrolyte interface. The MD simulation utilized a layered configuration (Figure 5a) consisting of the Cu substrate (orange box), the UiO‐66‐NH_2_ overlayer (green box), and the overlying bulk phase (red box). Notably, a distinct high‐concentration region of K^+^ ions (red zone in Figure 5b) was observed near the UiO‐66‐NH_2_ modified Cu surface, indicating that the UiO‐66‐NH_2_ overlayer facilitates K^+^ accumulation at the interface, which is consistent with the conclusions drawn from the in situ FT‐IR and Raman spectroscopic analyses. Furthermore, water molecules were found to permeate through the UiO‐66‐NH_2_ pores (Figure 5c), forming a continuous hydrogen‐bonding network that promotes proton transfer, an essential step for the hydrogenation of NO_3_^−^ and ^*^NO_x_ intermediates. Interestingly, slightly higher concentrations of NO_3_^−^ were detected within the UiO‐66‐NH_2_ pores and at the Cu surface than in the bulk electrolyte (Figure 5d), suggesting that the UiO‐66‐NH_2_ overlayer creates a locally enriched microenvironment for K^+^ and NO_3_^−^, which collectively facilitate ^*^H generation and NO_3_^−^ supply during eNO_3_RR. It should be noted that the UiO‐66‐X overlayer contains unavoidable interparticle gaps in addition to its intrinsic pore network. These gaps act with the intrinsic MOF pores, enabling NO_3_^−^ and H_2_O to access the Cu surface through multiple transport pathways and thereby securing adequate delivery of NO_3_^−^ and protons. To clarify the origin of K^+^ enrichment induced by UiO‐66‐NH_2_, DFT calculations were performed. Considering that the porous UiO‐66‐X framework contains abundant carboxylate groups capable of attracting cations, and that the amino functionality in UiO‐66‐NH_2_ is expected to further strengthen this interaction, we evaluated the binding between K^+^·H_2_O and the amino‐functionalized linker (BDC–NH_2_). We calculated the adsorption energy of the K^+^·H_2_O complex on the BDC–NH_2_ ligand, which was found to be exothermic (−0.81 eV, Figure 5e), indicating a strong binding affinity between K^+^ and the amino‐functionalized linker. This result provides a molecular‐level explanation for the experimentally observed preferential enrichment of K^+^·H_2_O at the UiO‐66‐NH_2_ modified interface, and highlights the role of ligand functionalization in tuning interfacial hydration environments.

Building on the insights obtained from in situ FT‐IR spectroscopy, in situ Raman spectroscopy, and MD simulations, which collectively indicate that the UiO‐66‐NH_2_ overlayer significantly enriches K^+^ at the Cu interface, we next focused on clarifying how K^+^·H_2_O contributes to promoting nitrate reduction. The hydrogenation of adsorbed ^*^NO_3_^−^ to ^*^NHO_3_ is generally regarded as the rate‐determining step in the nitrate reduction reaction, and we therefore evaluated the corresponding activation barrier. The optimized structures in Figure 5f,g illustrate the adsorption configurations of ^*^NO_3_^−^ and ^*^NHO_3_^−^ on the Cu surface without and with interfacial K^+^, respectively. The calculated energy barrier for this step decreases significantly from 3.10 to 2.25 eV in the presence of K^+^ (Figure 5h), demonstrating that the K^+^·H_2_O complex facilitates proton activation and transfer, thereby accelerating the overall NO_3_^−^ reduction kinetics. In brief, MD simulations and DFT calculations reveal that the UiO‐66‐NH_2_ overlayer restructures the interfacial microenvironment by enriching K^+^·H_2_O complexes and facilitating proton activation, thereby accelerating NO_3_^−^ hydrogenation and enhancing overall ammonia selectivity.

## 3 Conclusion

In summary, we have developed a bio‐inspired interfacial hydration engineering strategy by integrating an amino‐functionalized UiO‐66 overlayer onto a Cu catalytic surface, enabling efficient and selective nitrate reduction to ammonia under neutral conditions. Under optimal conditions, UiO‐66‐NH_2_@Cu achieves a NH_3_ Faradaic efficiency of 98.6% with a high NH_3_ production rate of 5.02 mmol cm^−2^ h^−1^, corresponding to an ammonia partial current density exceeding 1 A cm^−2^. Combined in situ spectroscopy, MD simulations, and DFT calculations reveal that the UiO‐66‐NH_2_ overlayer reconstructs the interfacial water structure and promotes the local enrichment of reactive K^+^·H_2_O species. This hydration‐mediated microenvironment promotes the generation of active hydrogen species and effectively lowers the energy barrier for the rate‐determining protonation step, thereby accelerating the multi‐electron NO_3_^−^ reduction pathway toward NH_3_. Beyond nitrate reduction, this work highlights the critical function of interfacial water structure and hydrated cations in reaction kinetics, which provide valuable guidelines for designing catalysts for a wider range of proton‐demanding electrocatalytic reactions.

## Author Contributions

**Yuyin Mao**: conceptualization, investigation, writing – original draft, data curation, supervision. **Minghui Zhang**: conceptualization, writing – original draft, data curation. **Xiangdong Xue**: software, formal analysis, data curation. **Chengyu Guo**: writing – original draft, data curation. **Jian Liu**: resources, supervision, project administration, writing – original draft, conceptualization, funding acquisition.

## Conflicts of Interest

The authors declare no conflicts of interest.

## Supporting information




**Supporting File**: advs75361‐sup‐0001‐SuppMat.docx.

## Data Availability

The data that support the findings of this study are available in the supplementary material of this article.
