# Supplementary material for: Bioinspired Interfacial Hydration Engineering via Metal–Organic Frameworks for Efficient Nitrate‐To‐Ammonia Conversion in Neutral Media
Source: Adv Sci (Weinh). 2026 Apr 17;13(39):e75361. doi: 10.1002/advs.75361 (PMC13335440; doi:10.1002/advs.75361)
Supplement: Supplementary file 1 — Supporting File: advs75361‐sup‐0001‐SuppMat.docx. [file ADVS-13-e75361-s001.docx]

**Supporting Information**

**Bioinspired interfacial hydration engineering via metal–organic frameworks for efficient nitrate-to-ammonia conversion in neutral media**

*Yuyin Mao^[a]#^, Minghui Zhang^[a]#^, Xiangdong Xue^[a]^, Chengyu Guo^[a]^, Jian Liu^[a], [b]^**

[a] State Key Laboratory of Photoelectric Conversion and Utilization of Solar Energy, Qingdao New Energy Shandong Laboratory, Qingdao Institute of Bioenergy and Bioprocess Technology, Chinese Academy of Sciences, Qingdao 266101, P. R. China

[b] College of Materials Science and Engineering, Qingdao University of Science and Technology, Qingdao 266042, P. R. China

E-mail: [liujian@qibebt.ac.cn](mailto:liujian@qibebt.ac.cn)

**Methods**

**Chemicals and materials**

Potassium nitrate (KNO_3_), potassium hydroxide (KOH), potassium nitrite (KNO_2_), sodium hypochlorite (NaClO_3_), salicylic acid, sodium citrate and sodium nitroprusside (Na_2_[Fe(CN)_5_NO]·2H_2_O) were obtained from Beijing Chemical Corporation. Cu foil was purchased from Macklin Co. Nafion (5 wt%) was purchased from Macklin Co. All chemical reagents were used as received without further purifications. Deionized water was made by the Millipore system and used in all experiments.

**Synthesis of rCu**

Copper foils were immersed in 0.1 M HCl for 1 h to remove surface oxides, followed by ultrasonic cleaning in water and acetone for 30 min. The foils were then soaked in a mixed solution of 1 M KOH and 0.6 M sodium thiosulfate for 3 h to form CuO. The obtained CuO electrodes were subjected to cyclic voltammetry reduction in a three-electrode cell with KOH electrolyte, using a potential window of −0.8 to 0 V at a scan rate of 50 mV s⁻^1^. After rinsing with deionized water and ethanol, rCu was obtained.

**Synthesis of UiO-66-X@Cu electrodes**

UiO-66-X@Cu electrodes were prepared via electrochemical deposition based on previous studies.^[1, 2]^ Taking UiO-66 as an example, the precursor solution, which also served as the electrolyte, was prepared by dissolving 0.145 g ZrCl_4_ (6.25 mM), 0.21 g H_2_BDC (6.25 mM), 1.4 g LiNO_3_, and 0.9 mL H_2_O in 34 mL acetic acid and 100 mL DMSO. Using rCu as the working electrode, electrochemical deposition was performed in the precursor solution. Deposition was stabilized at 120 °C under an oil bath. Unless otherwise specified, the deposition time was 20 min at a potential of −1.6 V. Similarly, UiO-66-X was synthesized by replacing the organic linker H_2_BDC with 2-aminoterephthalic acid (BDC–NH_2_), 2-methylterephthalic acid, or 2-hydroxyterephthalic acid, while all other procedures remained identical.

**Characterizations**

The morphology and microstructure of the samples were characterized by SEM (Hitachi S-4800). The phase and structural information of the samples were detected by XRD (Bruker AXS D8 diffractometer). To ascertain the chemical composition and valence state, the samples were studied by XPS (Thermo Fisher Scientific Escalab 250 spectrometer). UV–vis absorption spectra were recorded by a Shimadzu UV 2550 spectrophotometer.

**Electrochemical NO_3_RR measurements**

All electrochemical measurements were carried out on a Chenhua 660 electrochemical workstation. An H-type cell separated by a bipolar membrane (BPM, FuMA FBM-PK) was employed to mitigate cathodic alkalization and maintain neutral reaction conditions. Prior to testing, the proton exchange membrane was pretreated by sequential immersion in 5% H_2_O_2_ at 80 °C for 1 h, rinsing with deionized water for 1 h, 0.5 M H_2_SO_4_ at 80 °C for 1 h, and a final rinse with deionized water for 1 h. Nitrate reduction reaction (NO_3_RR) experiments were conducted in a three-electrode setup in an H-type cell, utilizing Ag/AgCl as the reference electrode and platinum foil as the counter electrode, with potentials converted to the reversible hydrogen electrode (RHE) scale (E_RHE_ = E_Ag/AgCl_ + 0.198 + 0.0592pH). The electrolyte consisted of 0.5 M K_2_SO_4_ with 0.3 M NO_3_^–^ (KNO_3_), and all experiments were performed under an ultrahigh-purity N_2_ gas (99.99%) atmosphere. Prior to electrocatalytic testing, linear sweep voltammetry (LSV) curves were recorded at 10 mV s^−1^ from 0 to −1.3 V until steady-state polarization curves were achieved. Subsequent potentiostatic tests were conducted from −0.9 to −1.3 V vs. RHE (intervals of −0.1 V) for 1 hour to measure ammonia Faradaic efficiencies and yield rates. The electrocatalytic active surface area (ECSA) was calculated by analyzing the double-layer capacitance (C_dl_) extracted from cyclic voltammograms (CVs) measured at scan rates of 10-100 mV/s within the non-Faradaic potential region. The ECSA was then determined using the equation ECSA = C_dl_/Cs, where Cs represents the specific capacitance (0.04 mF).

The concentration of nitrate (NO_3_^–^), ammonia (NH_4_^+^) and nitrite (NO_2_^–^) were quantified by an ultraviolet-visible spectrophotometer (Shimadzu).

*Faradaic efficiency:* The selectivity of ammonia and nitrite products is determined using the following formulas:

FE (NH_3_) = (8 × F × *C* (NH_3_) × *V*) / *Q*

FE (NO_2_^–^) = (2 × F × *C* (NO_2_^–^) × *V*) / *Q*

Where (8, 2) are the number of electrons transferred for the formation of (NH_3_, NO_2_^–^), F is the Faraday constant (96485 C mol^–1^), C is the concentration of the product measured by UV-Vis, V is the volume of the electrolyte, and Q is the total charge.

**Stability testing**

Specifically, the stability test was conducted using a circulating electrolyte system. A large volume (1000 mL) of electrolyte containing 0.5 M K_2_SO_4_ and 0.3 M KNO_3_ was stored in a glass bottle. A peristaltic pump was used to continuously circulate the electrolyte between the glass bottle and the cathodic chamber of a H-cell reactor separated by a bipolar membrane (BPM) at a constant flow rate of 60 mL/min. During the long-term continuous electrolysis at a constant potential of −1.1 V vs. RHE, 1 mL of the electrolyte was periodically extracted from the 1 L reservoir to quantify the generated ammonia via UV-vis absorption spectroscopy.

**Determination of NH_3_**

Colorimetric method: In detail, 0.1 mL of electrolyte was taken from the electrochemical reaction vessel, diluted 200–800 times, and 4 mL of the diluted solution was taken for addition of 2 mL of 1 M NaOH solution containing 5 wt% salicylic acid and 5 wt% sodium citrate was, respectively. Then, 1 mL of 0.05 M NaClO and 0.2 mL of 1 wt% C_5_FeN_6_Na_2_O·2H_2_O were added into the above solution. After standing at room temperature for 2 h in dark condition, the UV–vis absorption spectrum was measured. The concentration absorbance curves were calibrated using standard ammonia chloride solution with a series of concentrations.

^1^H NMR methods: 0.5 mL of electrolyte was taken from the electrochemical reaction vessel. To inhibit the proton chemical exchange of ammonium ions, 100 μL of 0.1 M HCl was added to the sample. Subsequently, 0.5 mL of the pH-adjusted solution was mixed with 200 μL of DMSO-d^6^ as an internal standard. The mixture was then transferred into a standard 5 mm NMR tube and analyzed by 1H NMR spectroscopy (400 MHz). The concentration calibration curves were established by integrating the peak areas of standard NH_4_Cl solutions with a series of concentrations prepared under identical conditions.

**Determination of NO_2_^–^**

The color reagent was prepared by dissolving 0.04 g N- (1-naphthyl) ethylenediamine dihydrochloride, 0.8 g sulfamic acid, and 2 mL phosphoric acid in 10 mL water. 0.1 mL of electrolyte was taken from the electrochemical reaction vessel and diluted 10-fold to 1 mL. From this, 0.05 mL was mixed with 2 mL 1 M HCl and 50 μL of color reagent, left to stand for 30 min, and then analyzed by UV–vis spectroscopy. The concentration absorbance curves were calibrated using standard KNO_2_ solution with a series of concentrations.

**Determination of NH_2_OH**

The detection reagents were prepared by dissolving 19.3 mg of ferric ammonium sulfate in 10 mL water (4 mM) and 19.8 mg of 1,10-phenanthroline in 10 mL ethanol (10 mM), alongside an acetate buffer containing 1 M sodium acetate and 1 M acetic acid. 0.5 mL of electrolyte was taken from the electrochemical reaction vessel and diluted 10-fold to 5 mL. From this, 3 mL was mixed with 100 μL of acetate buffer, 100 μL of ferric ammonium sulfate solution, and 100 μL of 1,10-phenanthroline solution, left to stand for 30 min, and then analyzed by UV–vis spectroscopy at 510 nm. The concentration-absorbance curves were calibrated using standard NH_2_OH·HCl solution with a series of concentrations.

**Quasi *in situ* ESR measurement**

Hydrogen radicals (*H) were detected by electron spin resonance (ESR) using 5,5-dimethyl-1-pyrroline N-oxide (DMPO) as the spin-trapping agent. The electrolyte was purged with argon for 30 min to remove dissolved oxygen, followed by addition of 100 μL of 100 mM DMPO to 40 mL of electrolyte. After 5 min of electrolysis, the solution was collected and analyzed on a Bruker A300 ESR spectrometer at room temperature.

**K^+^ retention experiment**

K^+^ retention experiments were conducted in 0.3 M KNO_3_ using a standard three-electrode setup. The UiO-66-NH_2_@Cu electrode served as the working electrode, with a Pt counter electrode and an Ag/AgCl reference electrode. Immediately after immersion in the electrolyte, a constant potential of −2.01 V (vs Ag/AgCl) was applied. After 120 s of polarization, the working electrode was removed, briefly rinsed with 5 mL of deionized water, and the potential was released. The adsorbed K^+^ was subsequently desorbed into the rinsing water, and its concentration was quantified by ICP-OES.

***In situ* FT-IR spectroscopy**

*In situ* FT-IR was performed on the FT-IR spectrometer (Bruker) with liquid nitrogen cooled HgCdTe detector. A modified accessory at an incident angle was used, and the measurement was conducted in an electrochemical cell furnished with Pt foil and Ag/AgCl as counter and reference electrodes Before testing, the detector was cooled with liquid nitrogen for at least 30 min to maintain a stable signal. Chronoamperometry was used for NO_3_RR test and was accompanied by the spectrum collection (64 scans, 4 cm^−1^ resolution). All spectra were subtracted with the background.

***In situ* Raman spectroscopy**

*In situ* Raman measurements were conducted by employing a top-plate cell euphotic device with 0.3 M KNO_3_ and 0.5 M K_2_SO_4_ aqueous solution as the electrolyte. Raman spectra were collected using a Renishaw inVia microprobe Raman spectrometer, employing an excitation laser at 532 nm with 3 mW between 400 and 4000 cm^−1^.

**DFT method**

All structural optimizations and energy evaluations were carried out via spin-polarized density functional theory (DFT) within the CP2K package. The exchange–correlation effects were treated using the generalized gradient approximation with the Perdew–Burke–Ernzerhof (PBE) functional, supplemented with Grimme’s DFT-D3 empirical dispersion correction. Core electrons were represented by the Goedecker–Teter–Hutter (GTH) pseudopotentials, while valence electrons were expanded in a double-*ζ* valence polarization basis sets optimized for molecules and short-range (DZVP-MOLOPT-SR-GTH), minimizing basis set superposition errors. A plane‐wave cutoff of 600 Ry was applied for the auxiliary basis. Geometry optimizations were performed using the Broyden–Fletcher–Goldfarb–Shanno (BFGS) algorithm, with forces converged to within 4.5×10^−4^ Hartree/Bohr. Thermodynamic properties were subsequently computed based on the optimized structures, and analyzed by Shermo software. The CP2K input file was generated using the Multiwfn.


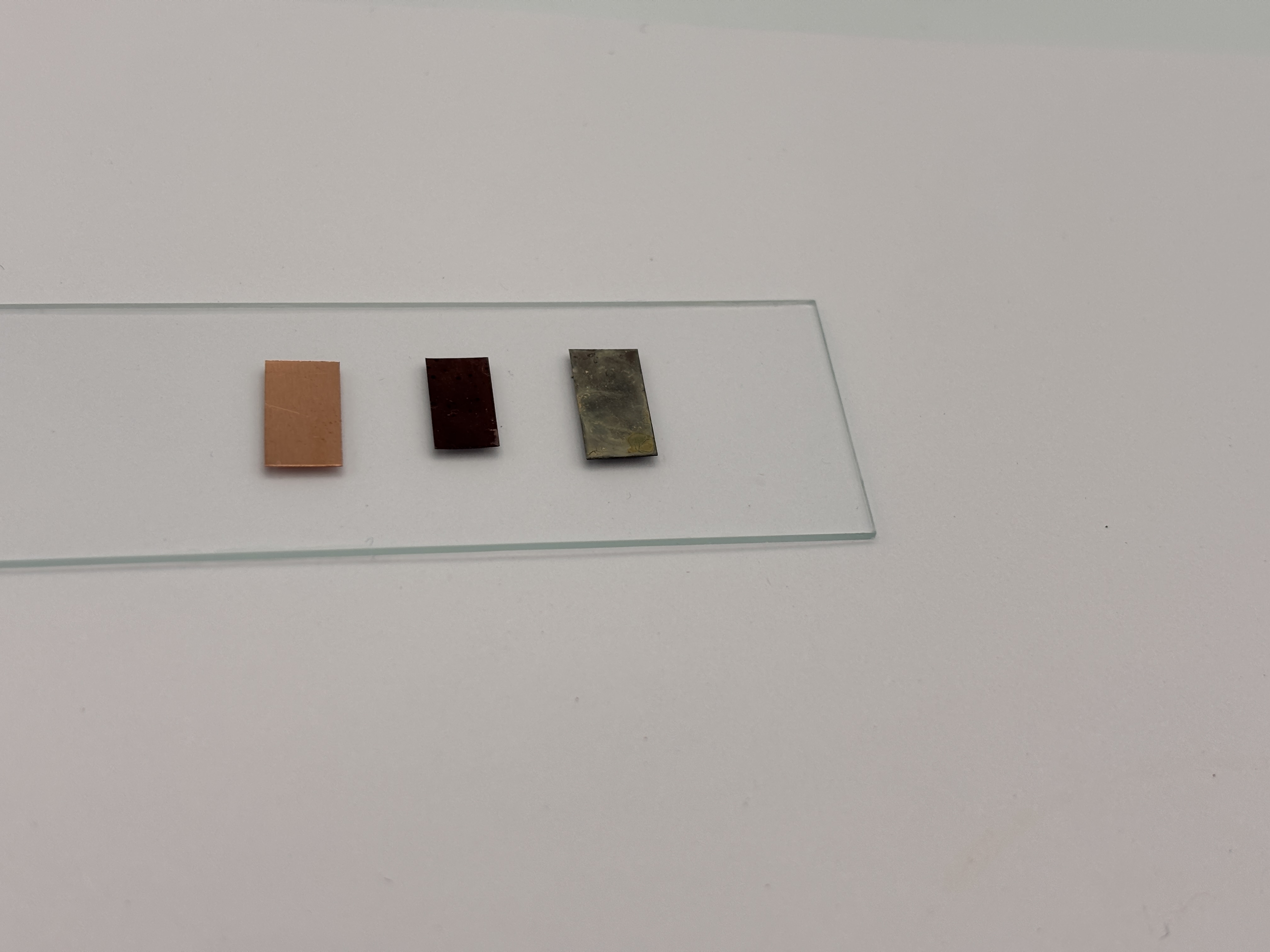


**Figure S1**. Digital images of the Cu foil, rCu, and UiO-66-NH_2_@Cu electrodes (from left to right).


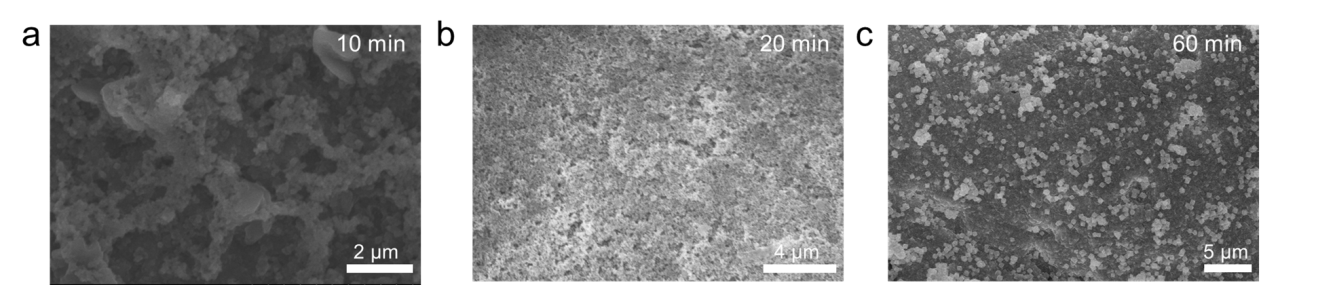


**Figure S2.** SEM images of UiO-66-NH_2_ overlayers deposited on Cu substrates at different electrochemical deposition times: (a) 10 min, (b) 20 min, and (c) 60 min.


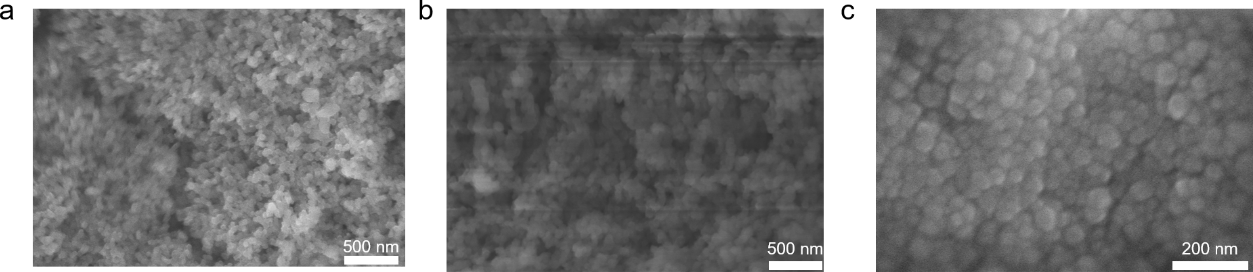


**Figure S3.** SEM images of (a) UiO-66@Cu, (b) UiO-66-OH@Cu, and (c) UiO-66-CH_3_@Cu.


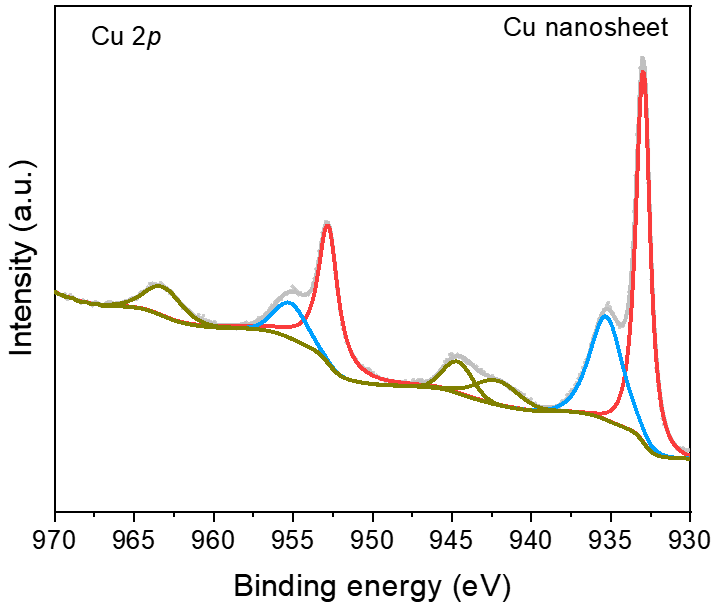


**Figure S4.** High-resolution Cu 2*p* XPS spectrum of the rCu electrode.


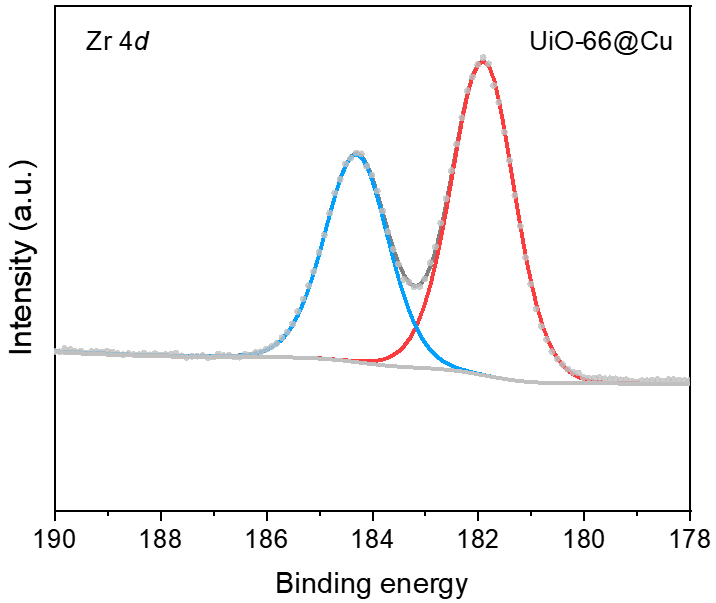


**Figure S5.** High-resolution XPS spectra of the UiO-66@Cu electrode in Zr 4*d* regions.


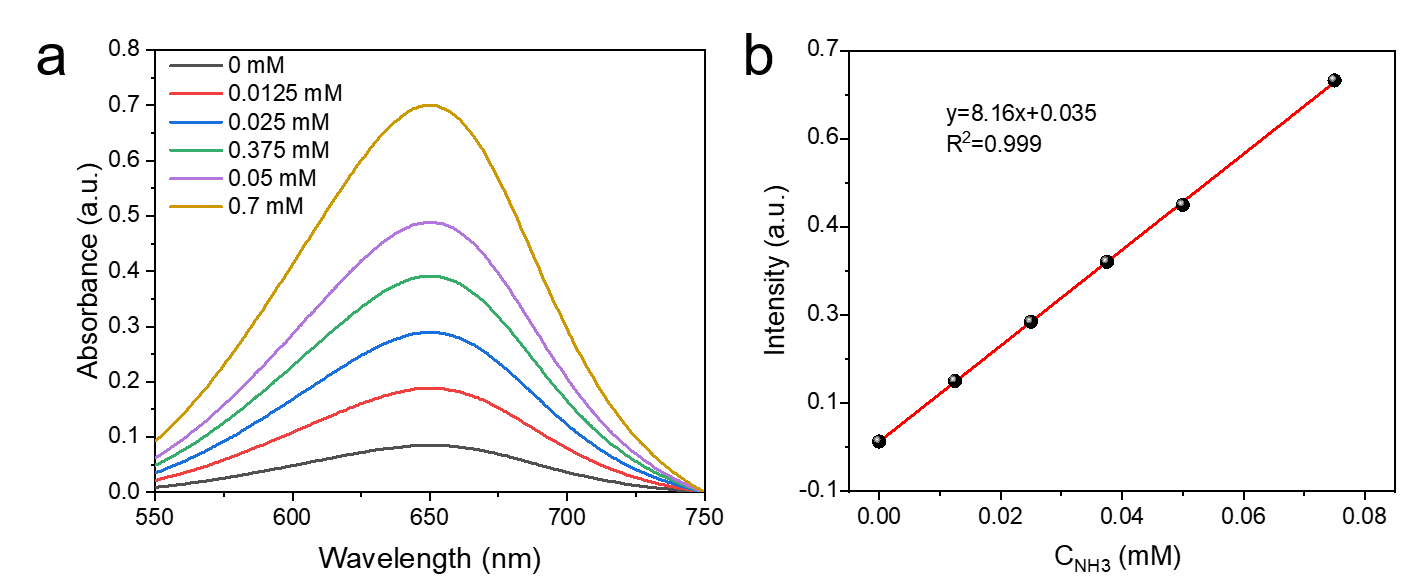


**Figure S6.** The absorption curves (a) and the corresponding concentration-absorbance calibration curve (b) of ammonia under 0.5 M K_2_SO_4_ conditions.


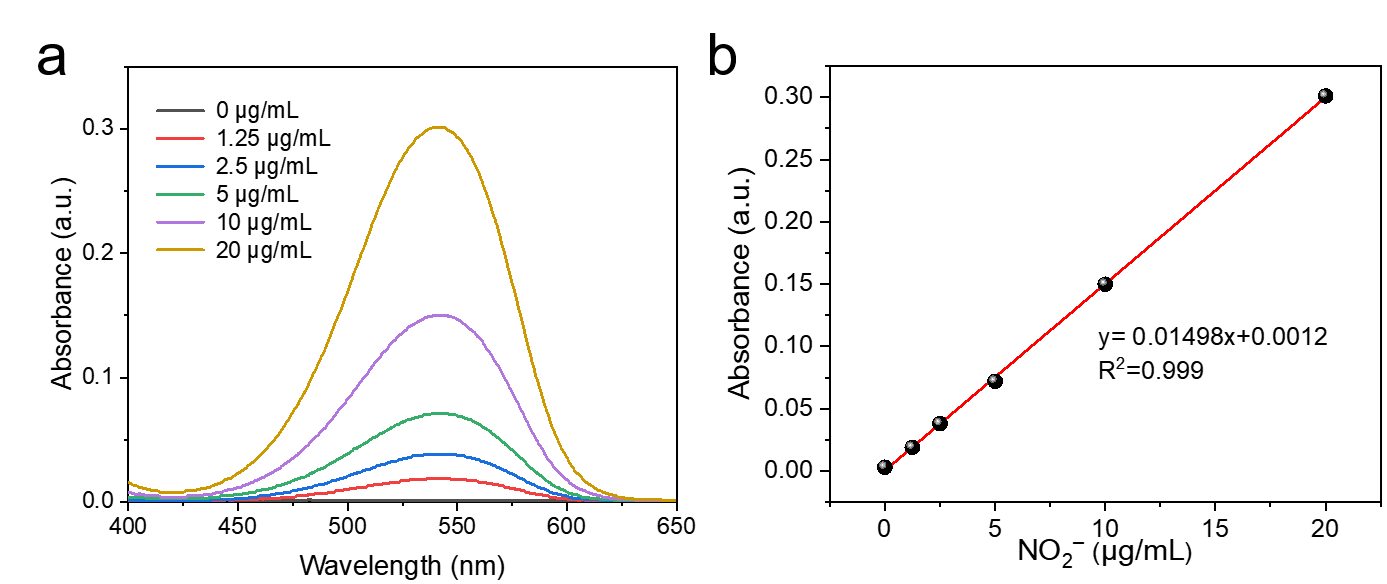


**Figure S7.** The (a) absorption curves and the corresponding (b) concentration-absorbance calibration curve of nitrite under 0.5 M K_2_SO_4_ conditions.


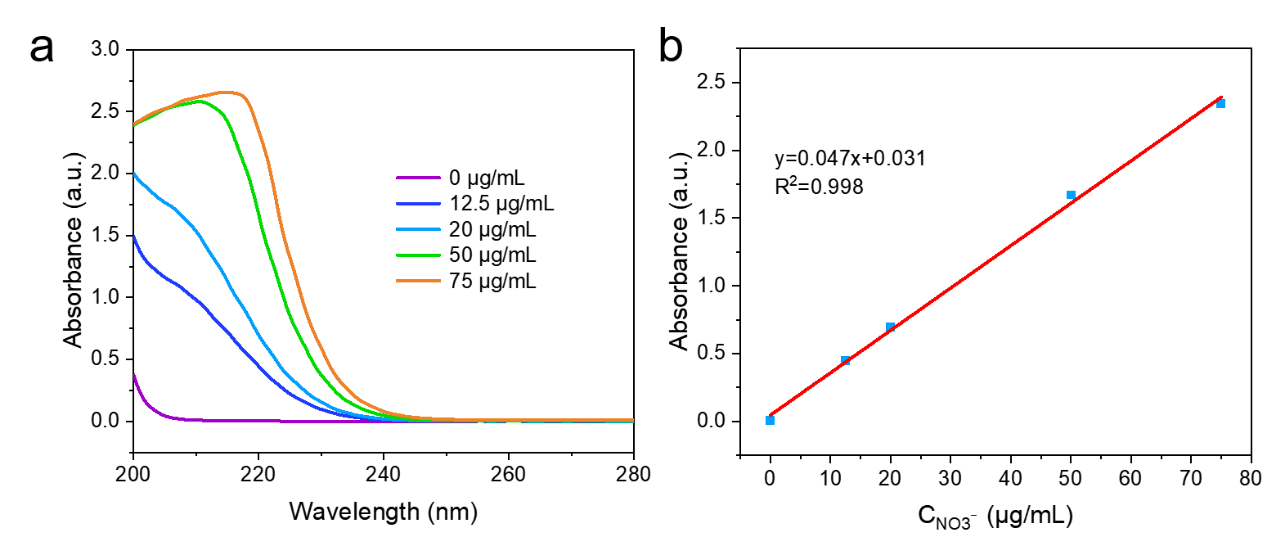


**Figure S8.** The (a) absorption curves and the (b) corresponding concentration-absorbance calibration curve of nitrate.


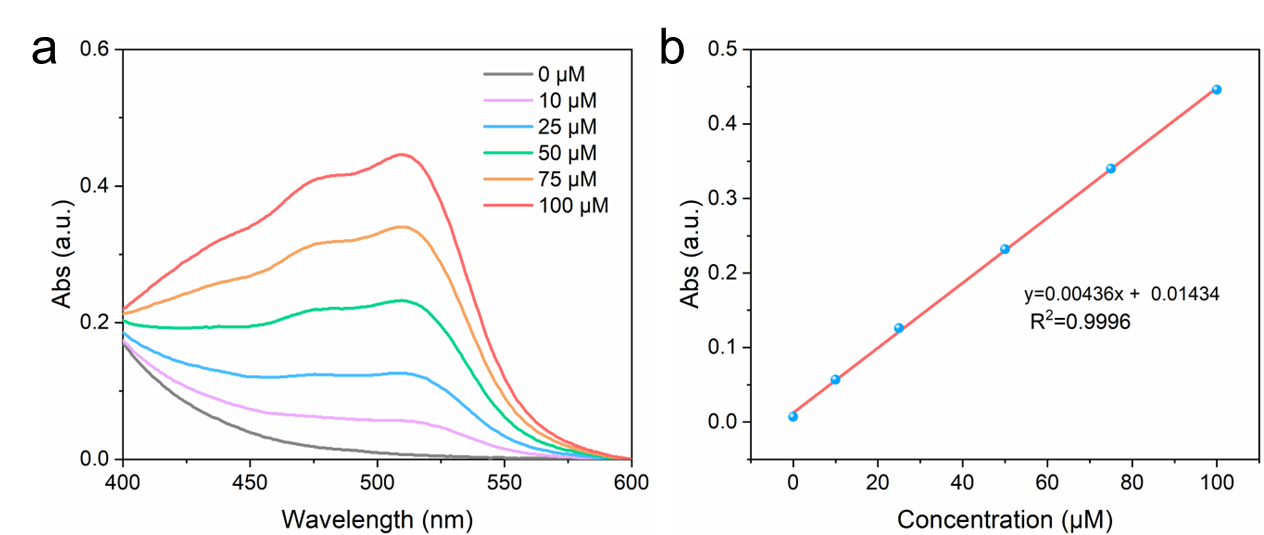


**Figure S9.** The (a) absorption curves and the (b) corresponding concentration-absorbance calibration curve of NH_2_OH.

**
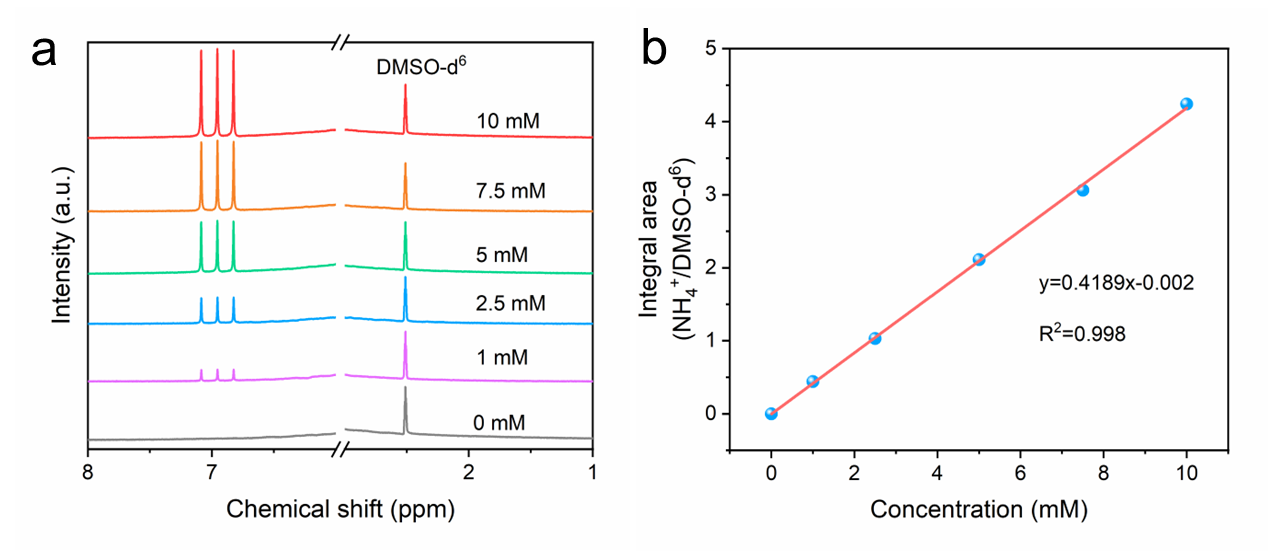
**

**Figure S10.** Detection and quantification of ^14^NH_4_^+^ by ^1^H NMR spectra (a) ^1^H NMR spectra of NH_4_^+^ with different concentrations. (b) The standard curve of integral area (NH_4_^+^ /DMSO-d^6^) against NH_4_^+^ concentration.


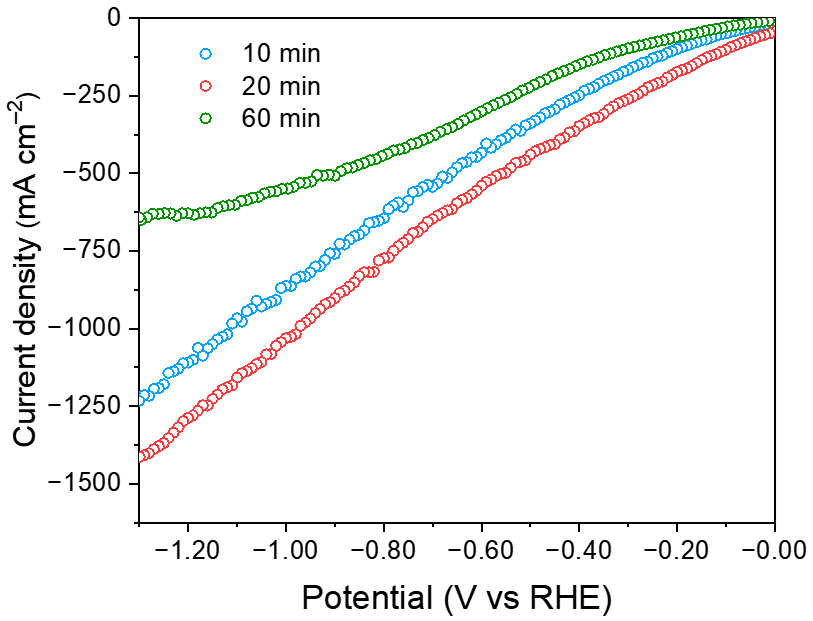


**Figure S11.** LSV curves of UiO-66-NH_2_ overlayers electrodeposited on Cu foils with different deposition times (10, 20, and 60 min) in 0.5 M K_2_SO_4_ electrolyte containing 0.3 M NO_3_^–^.

**
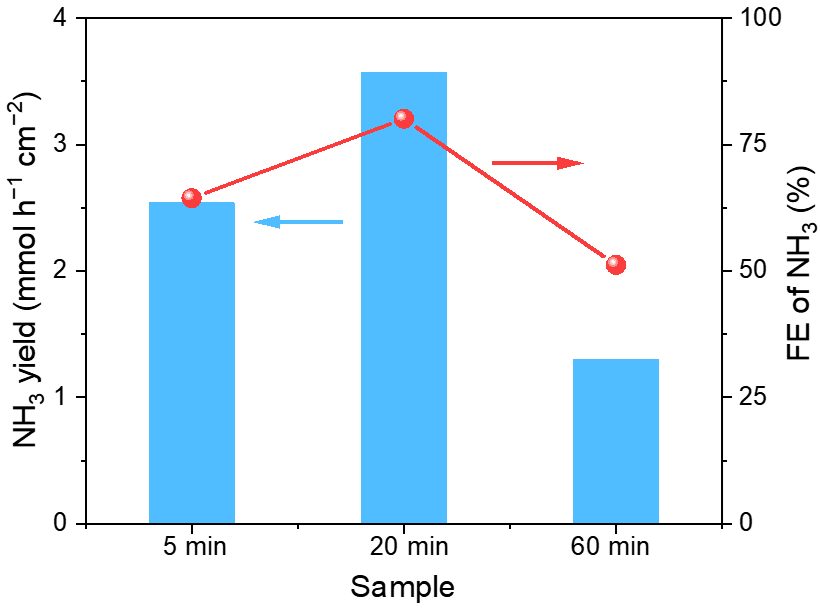
**

**Figure S12.** Optimal FE and yield for electrocatalytic nitrate reduction to ammonia of samples with different deposition times (10, 20, and 60 min).





Figure S13. NH_3_ yield and FE of UiO-66-NH_2_@C, rCu and UiO-66@Cu.


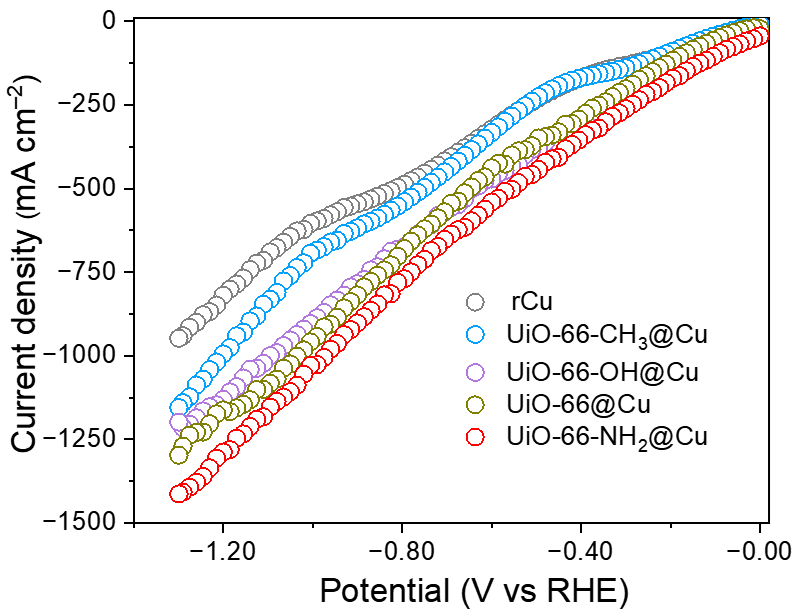


**Figure S14.** LSV curves of UiO-66-X@Cu in the presence of NO_3_^–^.


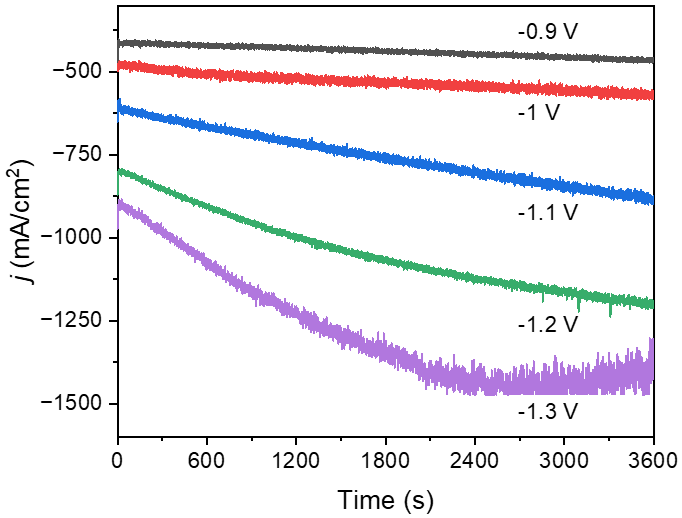


**Figure S15.** Chronoamperometric (i–t) curves of the rCu electrode recorded at various applied potentials.


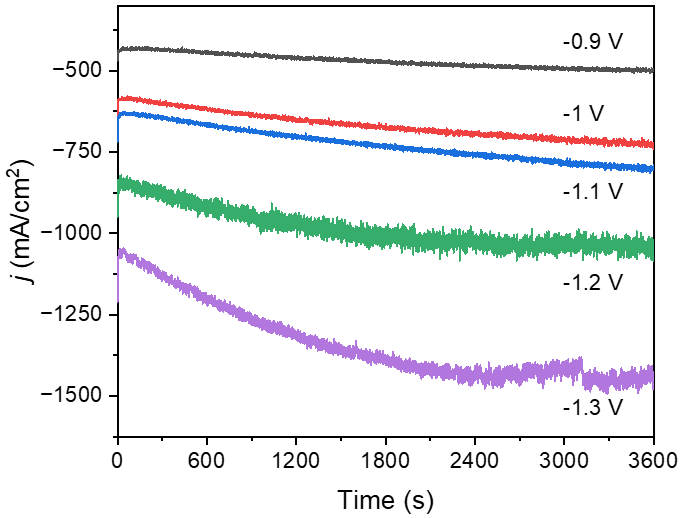


**Figure S16.** Chronoamperometric (i–t) curves of the UiO-66@Cu electrode recorded at various applied potentials.


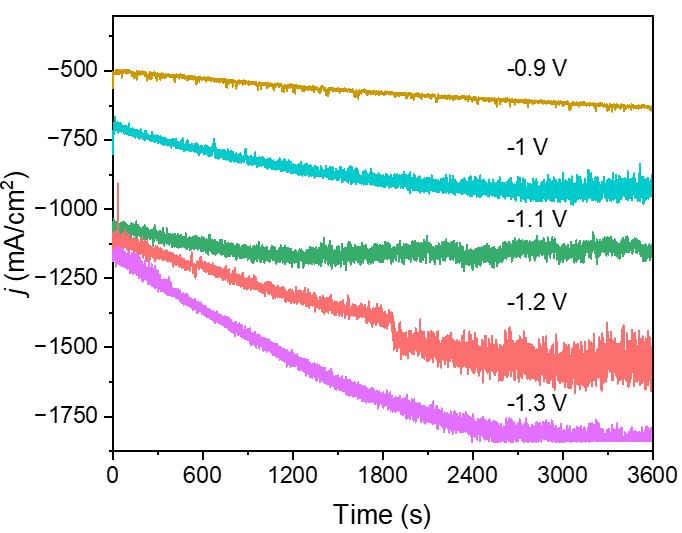


**Figure S17.** Chronoamperometric (i–t) curves of the UiO-66-NH_2_@Cu electrode recorded at various applied potentials.


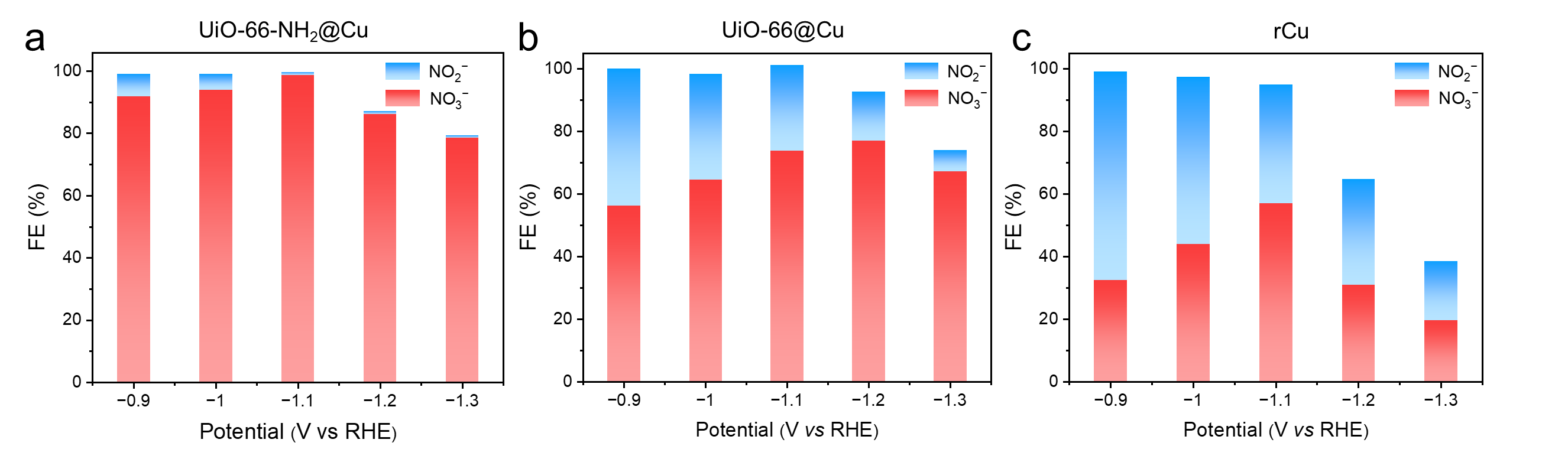


**Figure S18.** FE of (a) UiO-66-NH_2_@Cu, (b) UiO-66@Cu and (c) rCu under various potential.

**
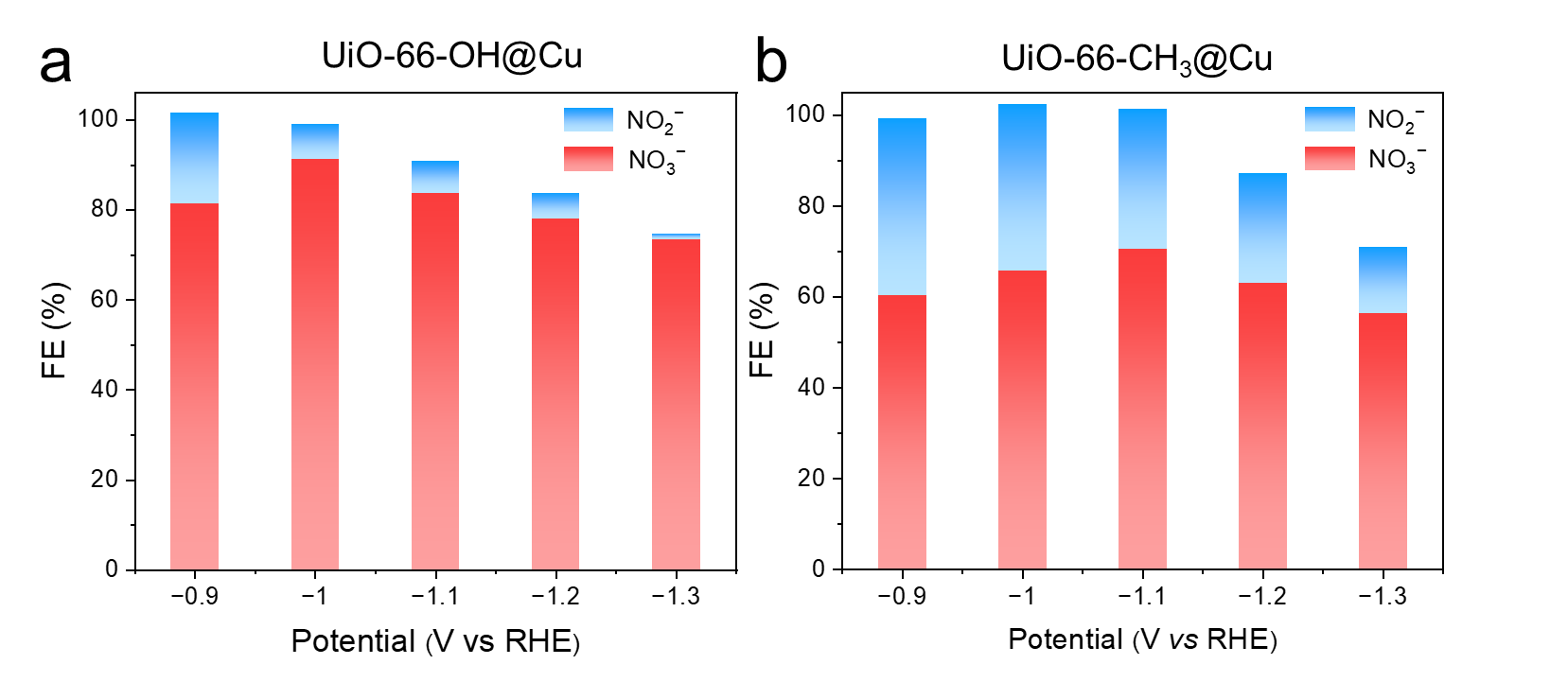
Figure S19.** FE of (a) UiO-66-OH@Cu and (b) UiO-66-CH_3_@Cu under various potential.

**
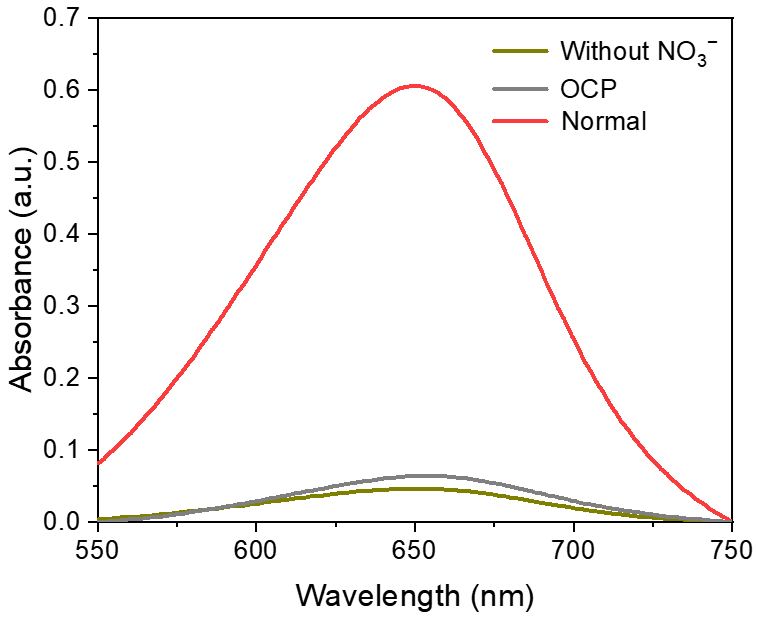
**

**Figure S20.** UV–vis absorption spectra of indophenol blue for NH_3_ quantification in control experiments.





**Figure 21.** ^1^H NMR spectra of electrolyte using ^14^NO_3_^−^ and ^15^NO_3_^−^ as reactants.

**

**

**Figure S22.** The yield of NH_4_^+^ determined by UV-vis and ^1^H NMR methods.





**Figure S23**. The UV-vis absorption spectra of the post-reaction electrolyte.


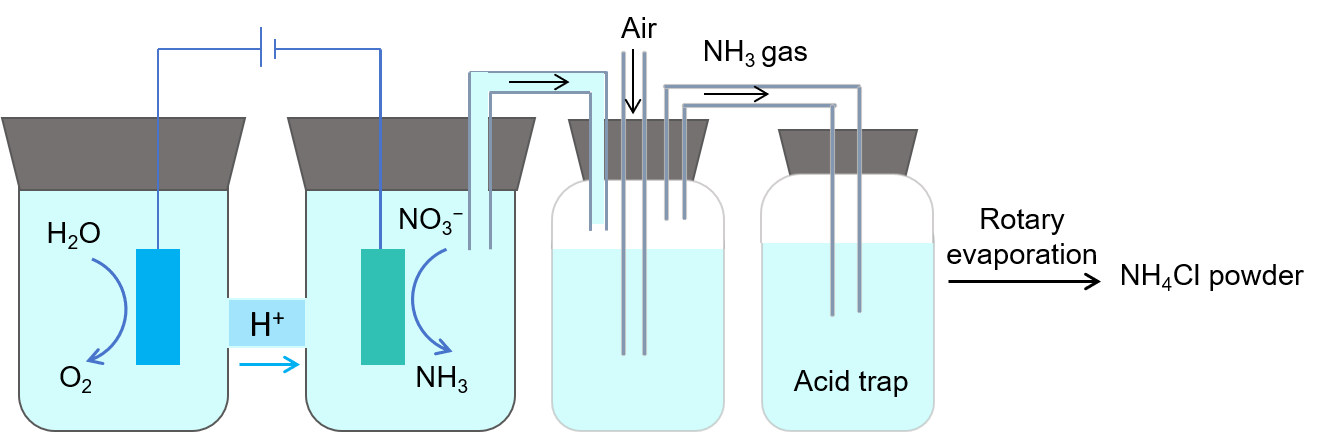


**Figure S24.** Schematic illustration of electrocatalytic nitrate reduction coupled with gas-stripping for the synthesis and collection of ammonium chloride powder.





**Figure S25.** XRD patterns of UiO-66-NH_2_@Cu electrode before and after reaction.


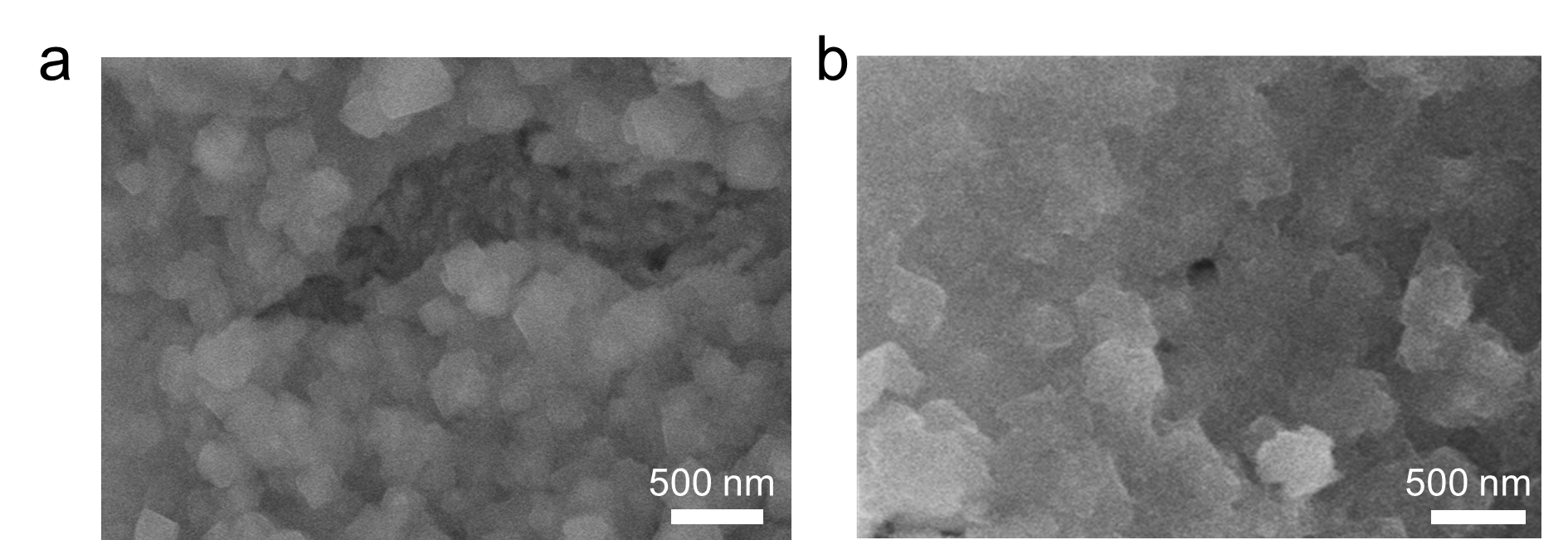


**Figure S26.** SEM images of UiO-66-NH_2_@Cu electrode (a) before and (b) after reaction.





**Figure S27.** FT-IR spectra of UiO-66-NH_2_@Cu electrode before and after reaction.


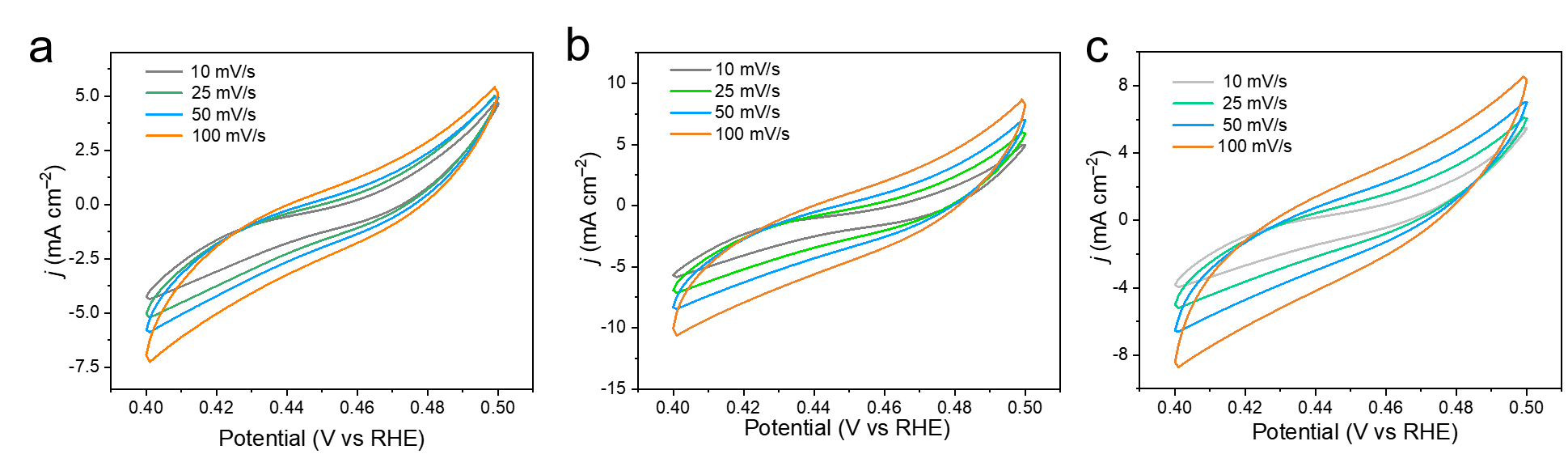


**Figure S28.** CV curves of (a) rCu and (b) UiO-66@Cu and, (c) UiO-66-NH_2_@Cu with different scan rates.


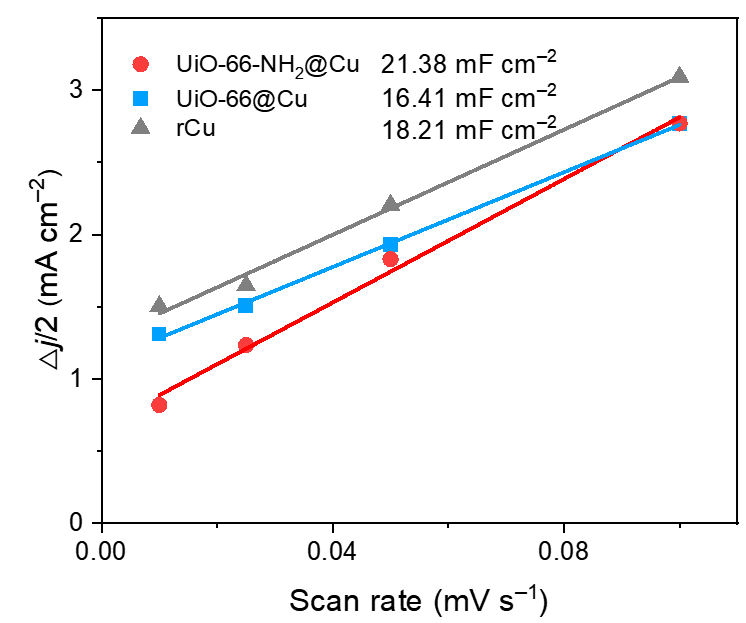


**Figure S29.** Corresponding plots of the current density versus the scan rates over three samples.


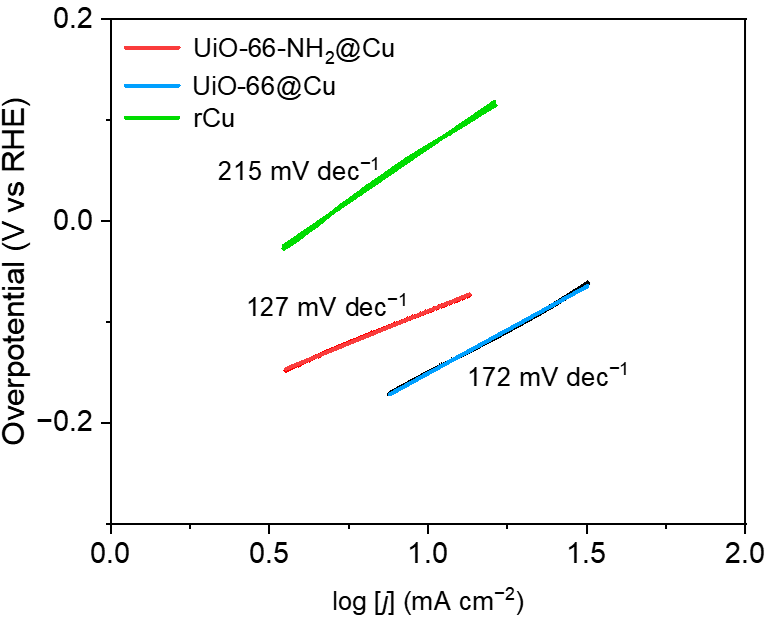


**Figure S30.** Tafel scope of rCu, UiO-66@Cu, and UiO-66-NH_2_@Cu.


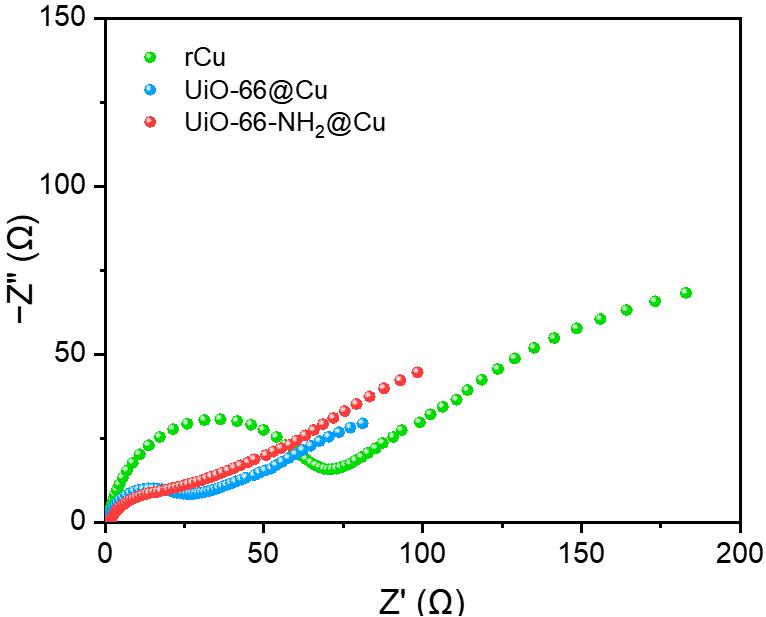


**Figure S31.** EIS Nyquist plots of rCu, UiO-66@Cu, and UiO-66-NH_2_@Cu.


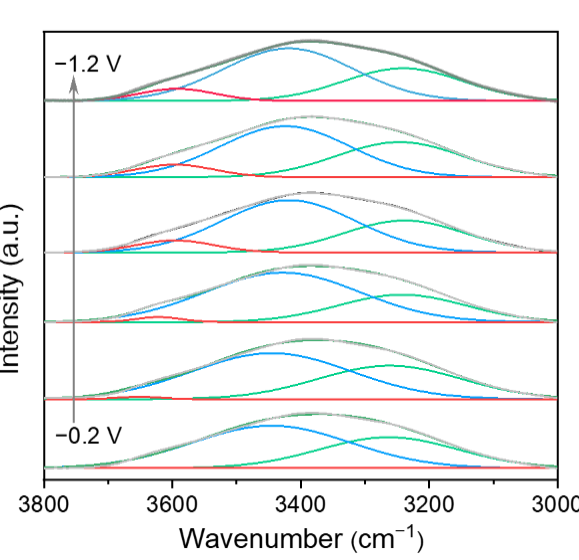


**Figure S32.** Gaussian-fitted peaks of *in situ* FT-IR spectra revealing the three O−H stretching modes of interfacial water on UiO-66@Cu in NO_3_RR process.


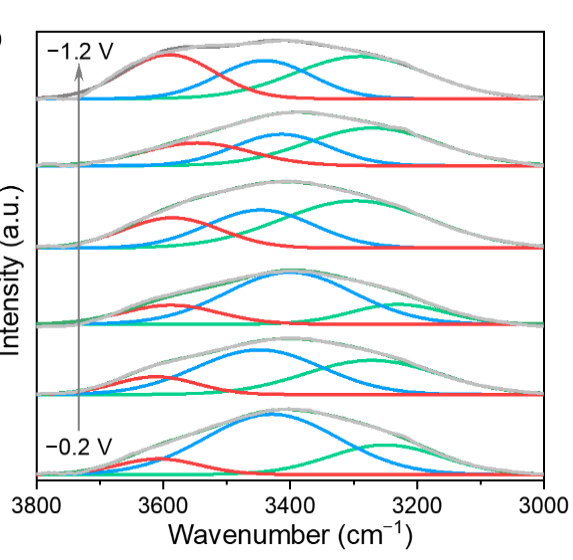


**Figure S33.** Gaussian-fitted peaks of *in situ* FT-IR spectra revealing the three O−H stretching modes of interfacial water on rCu in NO_3_RR process.


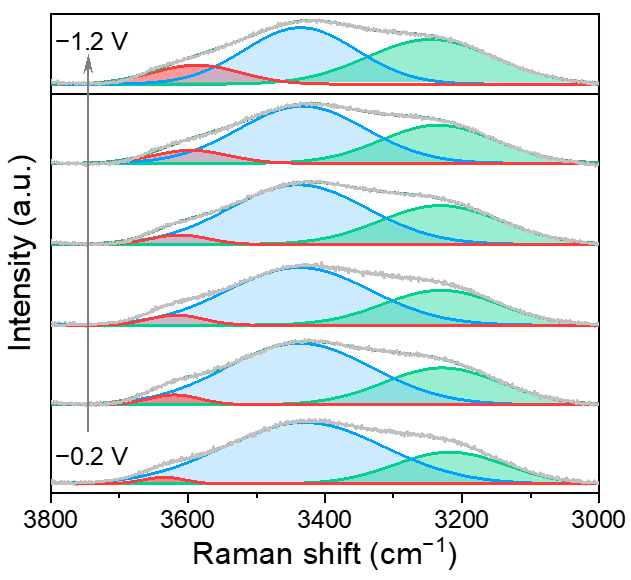


**Figure S34.** *In situ* Raman spectra of rCu under different potentials.


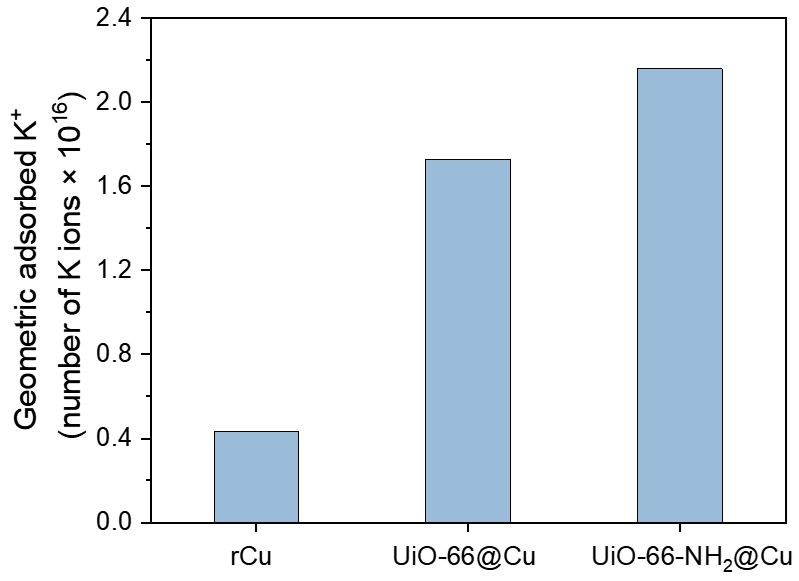


**Figure S35.** Geometric normalized of adsorbed K^+^ number on rCu, UiO-66@Cu and UiO-66-NH_2_@Cu electrodes.

**Table S1.** Comparison of NO_3_RR performances with the reported basic (gray) and neutral (green) NO_3_RR electrocatalysts.


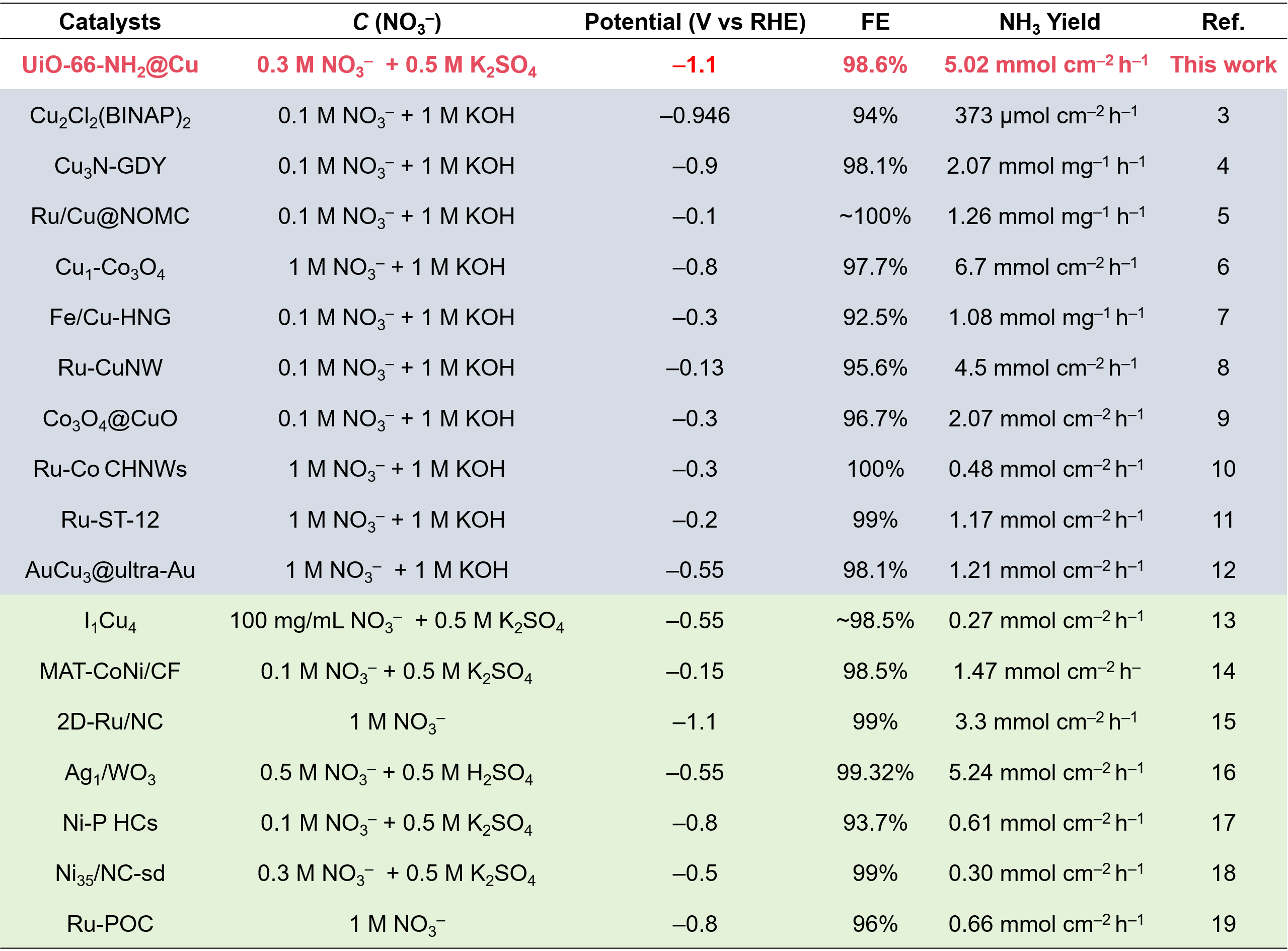


**Table S2.** The ICP testing data for Cu and Zr leaching of UiO-66-NH_2_@Cu after reacting in the electrolyte for 45 hours.


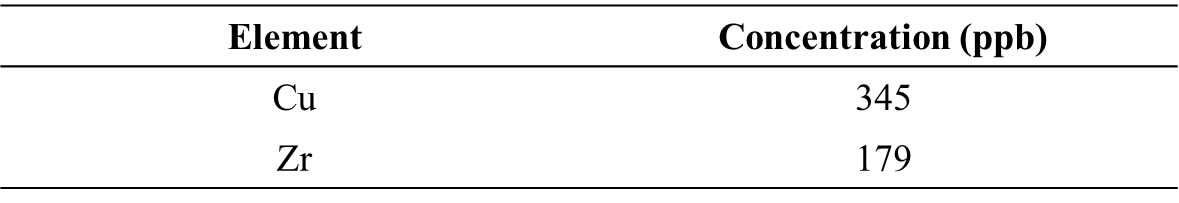


**Supporting References**

[1] S. Xie, W. Monnens, W. Zhang, W. Guo, N. Han, Z. Zhou, Z. Xue, I. F. J. Vankelecom, X. Zhang, J. Fransaer, *Cell Rep. Phys. Sci.* **2023**, *4*, 101412.

[2] S. Zhou, Y. Wei, L. Li, Y. Duan, Q. Hou, L. Zhang, L.-X. Ding, J. Xue, H. Wang, J. Caro, *Sci. Adv.*, **2018**, *4*, eaau1393.

[3] S.-J. Zheng, X.-Y. Dong, H. Chen, R.-W. Huang, J. Cai, S.-Q. Zang, *Angew. Chem. Int. Ed.* **2025**, *64*, e202413033.

[4] Z. Zhang, X. Feng, Z. Zhang, L. Chen, W. Liu, L. Tong, X. Gao, J. Zhang, *J. Am. Chem. Soc.* **2024**, *146*, 14898-14904.

[5] J.-J. Zhang, Y.-Y. Lou, Z. Wu, X. J. Huang, S.-G. Sun, *J. Am. Chem. Soc.* **2024**, *146*, 24966-24977.

[6] Y. Liu, J. Wei, Z. Yang, L. Zheng, J. Zhao, Z. Song, Y. Zhou, J. Cheng, J. Meng, Z. Geng, J. Zeng, *Nat. Commun.* **2024**, *15*, 3619.

[7] S. Zhang, J. Wu, M. Zheng, X. Jin, Z. Shen, Z. Li, Y. Wang, Q. Wang, X. Wang, H. Wei, J. Zhang, P. Wang, S. Zhang, L. Yu, L. Dong, Q. Zhu, H. Zhang, J. Lu, *Nat. Commun.* **2023**, *14*, 3634.

[8] F.-Y. Chen, Z.-Y. Wu, S. Gupta, D. J. Rivera, S. V. Lambeets, S. Pecaut, J. Y. T. Kim, P. Zhu, Y. Z. Finfrock, D. M. Meira, G. King, G. Gao, W. Xu, D. A. Cullen, H. Zhou, Y. Han, D. E. Perea, C. L. Muhich, H. Wang, *Nat. Nanotechnol.* **2022**, *17*, 759-767.

[9] H. Xu, Y. Yang, A. Han, C. Yao, H. Zhang, Y. Luo, Z. Fu, Y. Lu, G. Liu, F. Li, D. Zhao, *Angew. Chem. Int. Ed.* **2025**, *n/a*, e202510450.

[10] H. Cao, B. Liang, S. Ye, Z. Zhuang, L. Zheng, W. Chen, W. Chen, J. Zhang, Q. Zhang, J. Liu, X. Yan, Q. Zhang, *Chem. Eng. J.* **2024**, *490*, 151883.

[11] J. Li, G. Zhan, J. Yang, F. Quan, C. Mao, Y. Liu, B. Wang, F. Lei, L. Li, A. W. M. Chan, L. Xu, Y. Shi, Y. Du, W. Hao, P. K. Wong, J. Wang, S.-X. Dou, L. Zhang, J. C. Yu, *J. Am. Chem. Soc.* **2020**, *142*, 7036-7046.

[12] Y. Xiao, X. Tan, B. Du, Y. Guo, W. He, H. Cui, C. Wang, *Angew. Chem. Int. Ed.* **2024**, *63*, e202408758.

[13] B. Zhou, Y. Tong, Y. Yao, W. Zhang, G. Zhan, Q. Zheng, W. Hou, X.-K. Gu, L. Zhang, *Proc. Natl. Acad. Sci. U.S.A.* **2024**, *121*, e2405236121.

[14] Y. Wei, J. Huang, H. Chen, S.-J. Zheng, R.-W. Huang, X.-Y. Dong, L.-K. Li, A. Cao, J. Cai, S.-Q. Zang, *Adv. Mater.* **2024**, *36*, 2404774.

[15] S.-N. Zhang, P. Gao, Q.-Y. Liu, Z. Zhang, B.-L. Leng, J.-S. Chen, X.-H. Li, *Nat. Commun.* **2024**, *15*, 10877.

[16] J. Yu, R.-T. Gao, S. Ren, N. T. Nguyen, L. Wu, L. Wang, *Angew. Chem. Int. Ed.* **2025**, *n/a*, e202507696.

[17] Q. Hu, C. Shang, X. Chen, S. Qi, Q. Huo, H. Yang, C. He, *J. Am. Chem. Soc.* **2025**, *147*, 12228-12238.

[18] P. Gao, Z.-H. Xue, S.-N. Zhang, D. Xu, G.-Y. Zhai, Q.-Y. Li, J.-S. Chen, X.-H. Li, *Angew. Chem. Int. Ed.* **2021**, *60*, 20711-20716.

[19] G.-Y. Zhai, Q.-Y. Li, S.-N. Zhang, D. Xu, S.-Y. Xia, P. Gao, X. Lin, Y.-X. Lin, J.-H. Cheng, W.-Y. Hu, L.-H. Sun, X.-H. Li, J.-S. Chen, *CCS Chem.* **2022**, *4*, 3455-3462.
